# Principal process analysis of biological models

**DOI:** 10.1186/s12918-018-0586-6

**Published:** 2018-06-14

**Authors:** Stefano Casagranda, Suzanne Touzeau, Delphine Ropers, Jean-Luc Gouzé

**Affiliations:** 1 Université Côte d’Azur, Inria, INRA, CNRS, UPMC Univ Paris 06, Biocore team, Sophia Antipolis, France; 2 Univ. Grenoble Alpes, Inria, Grenoble, 38000 France; 3 Université Côte d’Azur, INRA, CNRS, ISA, Sophia Antipolis, France

**Keywords:** Dynamical systems, Biological networks, Process analysis, Model reduction, Parameter sensitivity analysis, Circadian clock

## Abstract

**Background:**

Understanding the dynamical behaviour of biological systems is challenged by their large number of components and interactions. While efforts have been made in this direction to reduce model complexity, they often prove insufficient to grasp which and when model processes play a crucial role. Answering these questions is fundamental to unravel the functioning of living organisms.

**Results:**

We design a method for dealing with model complexity, based on the analysis of dynamical models by means of Principal Process Analysis. We apply the method to a well-known model of circadian rhythms in mammals. The knowledge of the system trajectories allows us to decompose the system dynamics into processes that are *active* or *inactive* with respect to a certain threshold value. Process *activities* are graphically represented by *Boolean* and *Dynamical Process Maps*. We detect model processes that are *always inactive*, or *inactive* on some time interval. Eliminating these processes reduces the complex dynamics of the original model to the much simpler dynamics of the core processes, in a succession of sub-models that are easier to analyse. We quantify by means of global relative errors the extent to which the simplified models reproduce the main features of the original system dynamics and apply global sensitivity analysis to test the influence of model parameters on the errors.

**Conclusion:**

The results obtained prove the robustness of the method. The analysis of the sub-model dynamics allows us to identify the source of circadian oscillations. We find that the negative feedback loop involving proteins PER, CRY, CLOCK-BMAL1 is the main oscillator, in agreement with previous modelling and experimental studies. In conclusion, Principal Process Analysis is a simple-to-use method, which constitutes an additional and useful tool for analysing the complex dynamical behaviour of biological systems.

## Background

Mathematical modelling has been used for decades as an approach to understand the functioning of biological systems in terms of their internal processes and components. The latter form complex networks that vary in nature. For instance, biochemical networks include processes controlling the intracellular level of metabolites, RNAs and proteins, which allow cells to live and grow. A process either corresponds to a single biochemical reaction, for example protein phosphorylation, or encompasses many biochemical reactions like those involved in general cell functions (translation of proteins, transcription of RNAs...). In ecological networks, the processes can refer to events influencing the distribution and abundance of organisms, or to fluxes of energy and matter.

Numerous kinetic models of these networks have been developed in computational biology, of increasing complexity due to advances in modelling and parameter estimation approaches (see [1, 2] for an example). Complexity arises from the high dimension of the networks, the large number of biological processes involved and their non linearity due to the complex feedback loops that regulate them.

One approach often used to tackle the problem of complexity is model reduction (see [3] for a recent review). The simplified models are easier to analyse, while retaining the main features of the original ones and their biological significance. Briefly, methods of model reduction shorten the list of network species or of network reactions (e.g. [4, 5]), lump state variables (e.g. [6]) or decompose the system into slow and fast dynamics (e.g. [7–9]). The often used quasi-steady-state approximation falls in the latter category (e.g. [10]). Other approaches simplify the mathematical functions describing the molecular processes. For instance, piece-wise affine differential equations approximate by step functions the sigmoidal functions used to describe the regulation of gene expression. The dynamics of the simplified system can be easily analysed by means of state transition graphs [11]. However, these simplifications are generally restricted to models of gene expression and are more difficult to apply to other types of networks [12].

Reduction approaches have proven successful to significantly reduce model complexity, but they do not provide a mean to understand how the system dynamics emerges from the cascade of biological processes and regulatory mechanisms at work. This is especially true when the reduced models remain complex, with many coupled equations sharing common processes and involving complex feedback loops. For instance, regulatory mechanisms switch on certain biological processes at some times and off at others. It is thus important for a good understanding of the system behaviour to identify which and when processes significantly influence the system dynamics. In other words, instead of analysing a single reduced model in place of the original one, valid on the whole time interval, we may want to analyse series of simplified models highlighting the important processes of the original model during the periods of time in which they are *active*.

This is how we address the problem of high dimensional model analysis in this study. We develop a mathematical and numerical approach based on the boolean concept of *activity/inactivity*. The method, called *Principal Process Analysis* (PPA), determines the contribution of each biological process to the output of the dynamical system. In models of biological networks, these processes appear in a linear additive manner in each ODE. We first identify the *inactive* processes and neglect them. In a second step, we treat processes whose *activity* varies along time: we define time windows in which these processes are either always *active* or always *inactive*. We eventually create sub-models for each time window that only contain the *active* processes. This procedure leads to the simplification of the system to its core mechanisms. The simplified system can be further studied, to understand the role of each *active* process in the system dynamics.

PPA is a general approach that can be easily applied to any biological system described by ordinary differential equations (ODEs). It shares common features with a model reduction method focusing on major model parameters rather than processes [4], in which parameters that are not required for the system behaviour are removed. Another approach dedicated to chemical reactions identifies and removes chemical species that contribute less to the model output [5]. In this case, the problem is solved using optimization approaches (see also [13]). Despite these similarities, PPA is not a model reduction approach. It provides a mean to access to and dissect the more complex dynamics of the original model through the analysis of simplified versions in given time windows. Results are easily interpretable and do not require additional and complicated computations.

Preliminary work on PPA has been described in an earlier conference paper [14], in which we applied PPA to two ODE models of biochemical networks whose simplification preserved their dynamical behaviour: the model of circadian rhythms in *Drosophila* [15] and the model of the regulation of the ERK signalling pathway [16]. Questions remained open though, concerning the scalability of the approach and its robustness: to which extent does PPA preserve model dynamics in systems of higher dimension, with many more biological processes involved and including interlocked feedback loops? And since the approach requires a priori knowledge of the parameter values, how sensitive are process *activities or inactivities* to the value of these parameters? In this study, we address these questions by studying a much more complex model of circadian rhythms in mammals, including 16 variables, 76 processes, and intertwined positive and negative feedback loops [17]. Parameter sensitivity analysis of the global relative error between the original and reduced systems allows us to assess the quality and robustness of our approach.

The paper is organized as follows. “Methods” section describes the principle of Principal Process Analysis as well as global sensitivity analysis. “Model description” section introduces the model of mammalian circadian clock. We apply our approach to this complex model in “Principal Process Analysis of the circadian clock model” to “Influence of parameter values” sections, before concluding in “Conclusions” section.

## Methods

We summarize below the basics of the method of Principal Process Analysis. We will use as running example the 14^*th*^ variable of the mammalian circadian clock model analysed in “Model description” section (see also Appendix B. It describes how the concentration of the nuclear form of protein BMAL1 (*B*_*N*_=*x*_14_) changes:

1 $$ {}\begin{aligned} \begin{array}{ll} \frac{dB_{N}}{dt}\,=\,&\,-\,V_{3B} \frac{B_{N}}{K_{p}+B_{N}}\,+\,\!V_{4B} \frac{B_{NP}}{K_{dp}+B_{NP}} \,+\,k_{5} B_{C} \,-\, k_{6} B_{N} \,-\, k_{7} B_{N} PC_{N}\\ &+k_{8} I_{N} - k_{dn} B_{N}. \end{array} \end{aligned}  $$

### Principal Process Analysis (PPA)

Consider the following ODE model of biological network:

2 $$  \dot{x}=f\left(x,p\right)  $$

where $x=\left (x_{1},x_{2},\ldots,x_{n}\right) \epsilon \mathbb {R}^{n}$ is the vector of component concentrations, $x0=\left (x0_{1},x0_{2},\ldots,x0_{n}\right) \epsilon \mathbb {R}^{n}$ the vector of their initial values and *p*$\epsilon \mathbb {R}^{b}$ the vector of parameters. Each equation is decomposed into a sum of biological processes:

3 $$  \dot{x}_{i}=\sum\limits_{j}f_{ij}\left(x,p\right)  $$

where *f*_*ij*_ represents the *j*^*th*^ process involved in the dynamical evolution of the *i*^*th*^ variable of the system over a period of time [ *t*_0_,*T*].

#### **Example 1**

Equation (1) includes seven processes, each associated with a specific biological function. They take a positive or negative value, depending on whether they affect positively or negatively the variation of BMAL1 concentration. The equation of the protein is rewritten as:

4 $$  \dot{x}_{14}= f_{14,1}+f_{14,2}+f_{14,3}+f_{14,4}+f_{14,5}+f_{14,6}+f_{14,7}  $$

where $f_{14,1}= - V_{3B} \frac {B_{N}}{K_{p}+B_{N}},...,f_{14,7}= - k_{dn} B_{N} $.

**Fig. 1:**
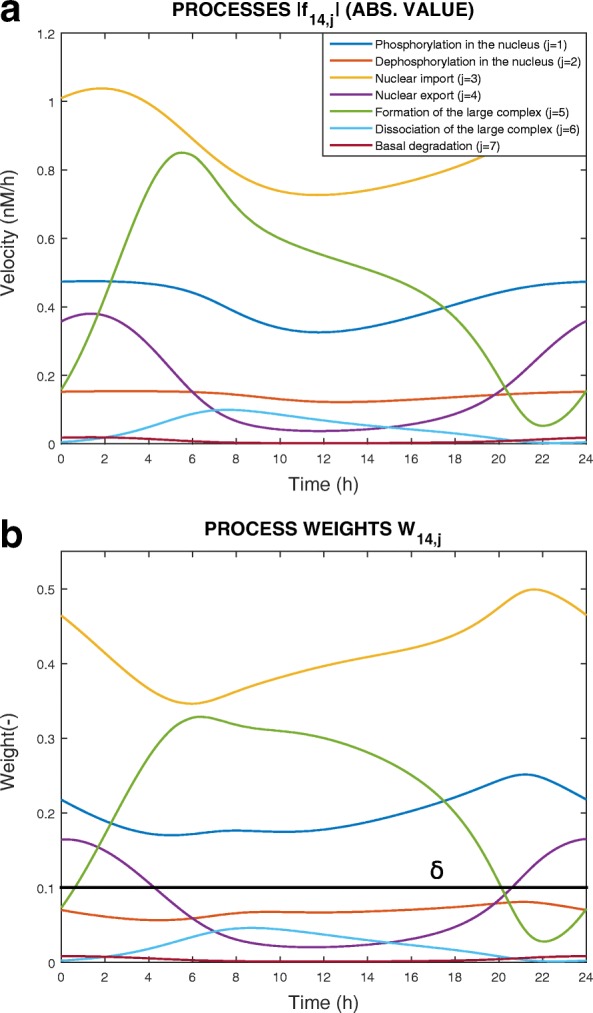
Dynamics of processes that change the nuclear concentration of protein BMAL1 (*B*_*N*_, see Eqs. (1) and (4)) over a 24-h time window. **a** Absolute value of the processes along time (one colour per process). **b** Weights associated with the processes along time. The threshold *δ* is set at 0.1

Figure 1a shows the dynamical evolution of processes *f*_14,1_ to *f*_14,7_ during a day. Nuclear import of BMAL1 is the fastest process of Eq. (1), while the basal degradation of the protein is the slowest.

Comparison criteria are needed to weigh the influence of the different processes *f*_*ij*_ on the time evolution of each variable *x*_*i*_. There are several alternatives. For instance, we can compare their absolute value (|*f*_*ij*_(*x*,*p*)|), scale it by the *i*^*th*^ initial condition $\left (\frac {|f_{ij}(x(t),p)|}{x_{0i}} \right)$, or scale it by the solution of the *i*^*th*^ ODE $\left (\frac {|f_{ij}(x(t),p)|}{x(t)_{i}}\right)$. In this work we associate a relative weight with each process to make it dimensionless:

5 $$  W_{ij}(t,p)=\frac{|f_{ij}(x(t),p)|}{\sum_{j}|f_{ij}(x(t),p)|}  $$

where 0≤*W*_*ij*_(*t*,*p*)≤ 1 and $\sum _{j}W_{ij}(t,p)=1$.

#### **Definition 1**

Let the continuous function *f*_*ij*_(*x*(*t*),*p*) be the *j*^*th*^ process of $\dot {x}_{i}(t)$ in *t* *ε*[*t*_0_,*T*] and let the threshold *δε* [0,1]. We call a process *f*_*ij*_(*x*(*t*),*p*) always inactive when *W*_*ij*_(*t*,*p*)<*δ*∀ t *ε* [0,T]. We call a process *f*_*ij*_(*x*(*t*),*p*) inactive at time *t* when *W*_*ij*_(*t*,*p*)<*δ*. We call a process *f*_*ij*_(*x*(*t*),*p*) active at time *t* when *W*_*ij*_(*t*,*p*)≥*δ*. Switching time for a process *f*_*ij*_(*x*(*t*),*p*) is the time $t_{ij}^{s}$ at which *W*_*ij*_(*t*,*p*)=*δ*. A process can have 0,1,…,*z* switching times. The switching time set *S*_*i*_ for the *i*^*th*^ variable contains all the switching times $t_{ij}^{s}$ where *j*=1,..,*k* and *s*=1,…,*z*. The global switching time set *S* is the union of all *S*_*i*_.

The choice of *δ* is important, since it determines above which weight a process can be considered *active or inactive* and, as we will see it later, if the process should be kept or omitted in the simplified model. An excessively high value might lead to an oversimplified model, without many dynamical features of the original model. Conversely a very low value might result in a model insufficiently simplified, which remains too complicated to analyse. From our experience, a convenient value is *δε* [0, 0.1], where the value of *δ* can be adjusted to the number of processes. For instance, if an ODE contains numerous processes of similar value, each individual process weighs little. In this case, *δ* should not be chosen too high to avoid omitting all these processes; it can be inversely proportional to the total number *N* of processes in the equation: $\delta \propto \frac {1}{N}$. In this paper, fine-tuning the threshold value is not justified: there are not many processes per equation and they have very different values. We will always take *δ*=0.1.

#### **Example 2**

We apply Eq. (5) to determine the dynamical weight of the seven processes in Eq. (1). Results are shown in Fig. 1b. As expected, the nuclear import, which is the fastest process, weighs more in the dynamical evolution of BMAL1 concentration, while the basal degradation of the protein weighs little. We determine the process activities using *δ*=0.1:

- The weight of processes *W*_14,2_, *W*_14,6_, *W*_14,7_ is always below *δ*: their related processes *f*_14,2_, *f*_14,6_, *f*_14,7_ are thus *always inactive*;
- The processes *W*_14,1_ and *W*_14,3_ are always above *δ*: *f*_14,1_ and *f*_14,3_ are *active* during the whole system dynamics;
- The weight of processes *W*_14,4_ and *W*_14,5_ crosses the threshold twice and the switching times $t_{14,4}^{1}=4.4$h, $t_{14,4}^{2}=20.7$h, $t_{14,5}^{1}=0.8$h and $t_{14,5}^{2}=20.3$h are collected in the set *S*_14_.

### Visualization of process *activities*

Graphical tools turn out to be useful to analyse the dynamical weights of complex systems such as the mammalian circadian clock model. We use three of them in PPA, which are described below.

**Fig. 2:**
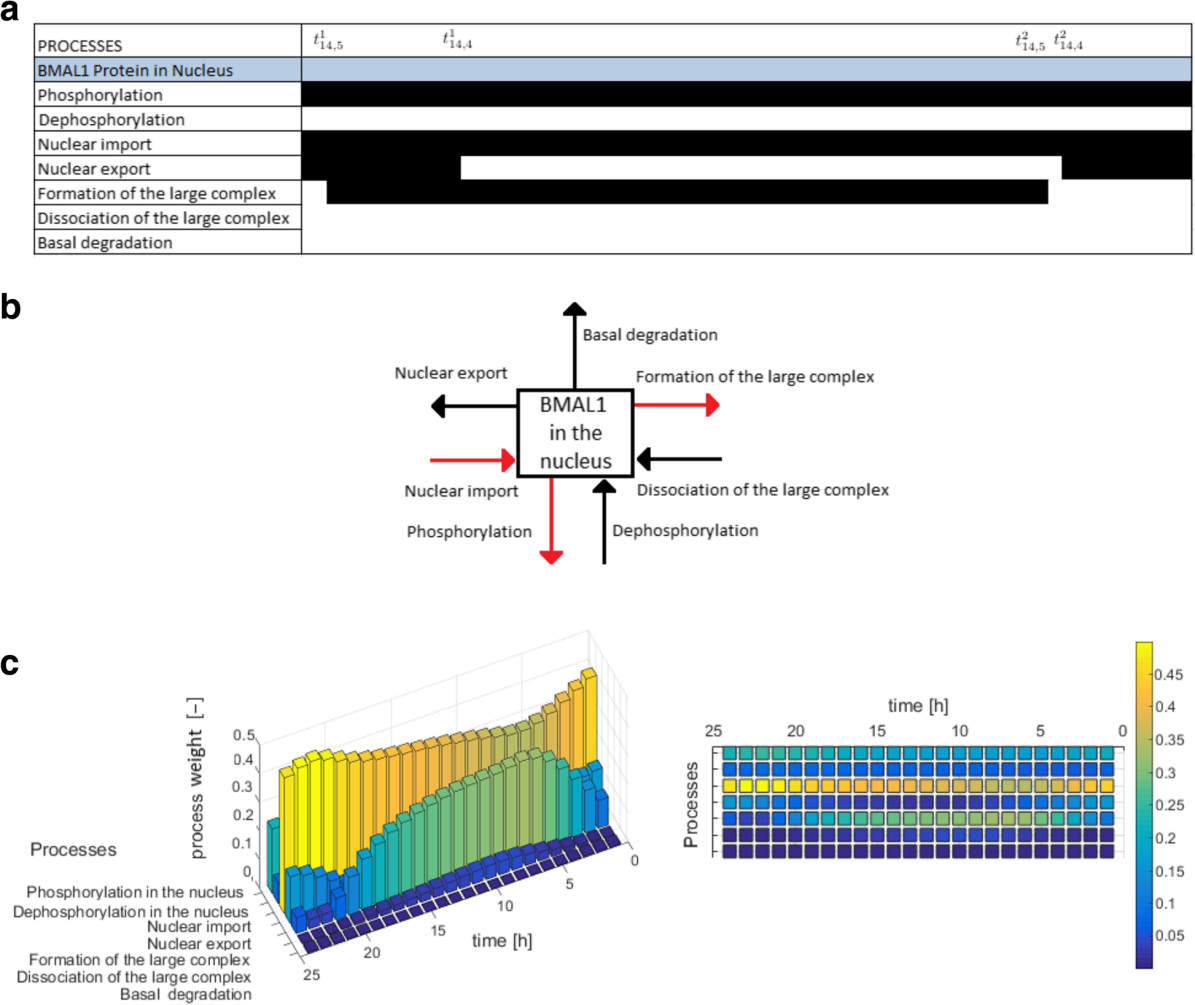
Visual tools. **a***Boolean Process Map*, **b***Dynamical Process Map* between times $t^{1}_{14,4}$ and $t^{1}_{14,5}$, **c***3-D Process Map* for the variable *x*_14_ and its corresponding 2-D version
- The *Dynamical Process Map* is a network representation of the process *activities*. Variables (represented by boxes) are connected by processes (arrows). Three cases arise, which depend on the *activity* of processes shared by several variables: black-coloured arrows represent processes that are *inactive* for all variables involved, while *active* processes are displayed as red arrows. Yellow arrows are used for processes shared by several variables that have different *activities*: for instance, one process is considered *active* in one equation, but *inactive* in another one. Note that the model simplification by elimination of *inactive* processes, as will be described in “Model simplification by elimination of *always inactive* processes” section, will have for effect to remove black arrows in the *Dynamical Process Map*.
- **Example 3** Figure *2*b represents the *Dynamical Process Map* for *x*_14_, the nuclear concentration of Bmal1, in the time interval between $t^{1}_{14,4}$ and $t^{1}_{14,5}$. Phosphorylation is an example of *active* process for the nuclear BMAL1 concentration (see the *Boolean Process Map* in Panel A). It is shown in red because it is also considered *active* at the same moment for the other variable sharing this process, the concentration of phosphorylated BMAL1.
- The *3-D Process Map* represents the time-dependent evolution of the intensity of each process. Process *activities* are averaged per hour, which leads to a discretisation of time. Vertical bars represent process weights for each hour. Their color code represents the intensity of process weights relatively to the other weights.
- **Example 4** Figure *2*c describes the *3-D Process Map* for the concentration of nuclear BMAL1. The phosphorylation of the protein, its nuclear import and its consumption for the formation of a large complex are the processes the most *active* over time.

- The *Boolean Process Map* summarizes qualitatively the knowledge of the process *activity* or *inactivity* along time for each variable. A black bar means that the process is *active*, while the white bar indicates an *inactive* process.
- **Example**: *The Boolean Process Map in Fig. *2a *represents the process activities deduced from the dynamical weights in Fig. *1b. *We observe that there is always an active phosphorylation of BMAL1 in the nucleus, while the basal degradation can be considered always inactive. The nuclear export is solely active in the first and last periods of time.*

### Model simplification by elimination of *always inactive* processes

Eliminating processes that play a minor role in the system dynamics facilitates the analysis of large models. Since in the previous steps of PPA we have determined the process *activities* in system (2), we now neglect processes that are considered *always inactive*. This will give us *g*(*x*^*r*^), the function approximating *f*(*x*) in (2) with less processes.

We thus introduce the ODE system (6), which approximates system (2):

6 $$  \dot{x}^{r}=g\left(x^{r},p^{r}\right)  $$

where $x^{r}=\left (x_{1}^{r},x_{2}^{r},\ldots,x_{n}^{r}\right) \epsilon \mathbb {R}^{n}$ is the vector of component concentrations, $x0=\left (x0_{1},x0_{2},\ldots,x0_{n}\right) \epsilon \mathbb {R}^{n}$ the vector of their initial values, and $p^{r} \epsilon \mathbb {R}^{c}$, where *c*≤*b* is the vector of parameters. The model simplification approach relies basically on the following theorem: if the vector fields of two systems are close (*f*(*x*)≈*g*(*x*)), then the solutions of the original and approximated systems are close during some time interval under the assumptions on the Lipschitz conditions listed in [18, p. 79, Th. 2.5].

Based on the dynamical weights determined in “Principal Process Analysis (PPA)” section and the threshold value *δ*, we apply the following rule to define *g*(*x*^*r*^,*p*^*r*^): *if* *W*_*ij*_(*x*(*t*),*p*) < *δ*∀ t *ε* [ *t*_0_,*T*]*theng*_*ij*_* = 0; if not, g*_*ij*_≡*f*_*ij*_.

We thus define *x*^*r*^ as an approximation of *x* and *p*^*r*^ as a subset of *p*.

#### **Example 5**

We proceed to the simplification of processes in Eq. 1. Because *f*_14,2_, *f*_14,6_, *f*_14,7_ are *always inactive*, *g*_14,2_=0, *g*_14,6_=0, *g*_14,7_=0 and *g*_14,1_≡*f*_14,1_, *g*_14,3_≡*f*_14,3_, *g*_14,4_≡*f*_14,4_, *g*_14,5_≡*f*_14,5_. The resulting ODE for $x^{r}_{14}$ is:

7 $$  \frac{dB^{r}_{N}}{dt}=-V_{3B} \frac{B^{r}_{N}}{K_{p}+B^{r}_{N}}+ k_{5} B^{r}_{C} - k_{6} B^{r}_{N} - k_{7} B^{r}_{N} PC^{r}_{N}.  $$

Note that Principal Process Analysis is applied to each ODE separately. As a consequence, processes shared by two equations can be *active* in one equation, but *inactive* in the other. Elimination of the *inactive* processes breaks mass balance in the simplified model. For our purpose, this is not an issue: the simplification does not aim at reducing the model, but rather analysing a sub-model of the original one, which describes the dynamics of the important phenomena.

It is interesting to quantify the extent to which the simplified system (6) preserves the behaviour of the original one. This gives a better sense of how the *active* processes kept in the simplified model are responsible for the dynamics of the original system. In addition, this helps identifying potential problems related to the model simplification, for instance involving a wrong choice of the *δ* value. One can also imagine pathological cases, when the simplified system does not reproduce the main dynamical features of the original model: for instance, if it evolves towards a different basin of attraction or if the removal of a consumption term does not compensate a synthesis term any more, leading the simplified system to explode in finite time. It is non nonsensical in all these cases to analyse simplified models that behave so differently from the original ones. The *δ* threshold should be adjusted to a new value and Principle Process Analysis re-run until model simplification proves satisfactory according to the criteria described below.

We present in Appendix A an a priori analysis of the error made when removing some *inactive* processes. This analysis gives a theoretical, but very conservative, bound on the error. In practice, we numerically compute the global relative error between the original and simplified models. Several forms of error are possible. We have chosen the following one, analysed over a period of time [ *t*_0_,*T*], in which *y*_*h*_ and $y^{r}_{h}$ are the *h*^*th*^ outputs of the original and simplified systems, respectively:

8 $$  e_{h}=\frac{\int_{t_{0}}^{T}|y_{h}(t)-y_{h}^{r}(t)|dt}{\int_{t_{0}}^{T}|y_{h}(t)|dt}.  $$

How to choose the model outputs? They can correspond to all model variables or combinations of them, if the latter are involved in some biological phenomena of interest for instance. In the case of the circadian clock model, six variables were specifically studied in the original papers [17, 19], which we will use as outputs to determine the global relative error between the original and simplified models: the concentrations of *Per* mRNA (*M*_*P*_), *Cry* mRNA (*M*_*C*_), *Bmal1* mRNA (*M*_*B*_), total PER protein (*P*_*Tot*_), total CRY protein (*C*_*Tot*_) and total BMAL1 protein (*B*_*Tot*_)^1^.

### Creation of sequences of sub-models

In the previous step of PPA, the models are simplified by elimination of *always inactive* processes. Here we go one step further in the simplification, by eliminating processes that are *inactive* at times. This is achieved by decomposing the period of time during which the system evolves into time intervals. To that end we use the *switching times* *t*_*b*_ (with *b*=1,…,*d*) determined in “Principal Process Analysis (PPA)” section: this allows creating a succession of sub-models for each time interval, which contain the core mechanisms in that period of time.

To avoid creating large sequences of sub-models, we reduce the number of time windows by grouping proximal *switching times* with the easy-to-compute k-means clustering [20]. Hence the *dswitching times* included in the *global switching time set* *S*=[*t*_1_,*t*_2_,...,*t*_*d*_] are grouped into *z* (≤*d*) clusters *C*= {*C*_1_,*C*_2_,...,*C*_*z*_}, so as to minimize the within-cluster sum of square (or within-cluster inertia):

9 $$  \mathrm{argmin_{C}}\sum\limits_{v=1}^{z}\sum_{t\epsilon C_{v}}||t-\mu_{v}||^{2}  $$

where *μ*_*v*_ is the mean of the *switching times* in *C*_*v*_. The consequence is that processes with *switching times* belonging to cluster *C*_*v*_ are assumed to switch together at the same time $t^{r}_{v}=\mu _{v}$, the mean *switching time* in cluster *C*_*v*_.

How to define the right number of clusters? A too large number of clusters will result in a low error, but also in numerous time windows that make the simplified models still too complex to analyse. Equation (10) describes how to take into account this trade-off between the number *z* of clusters and the error. It is related to the difference between the maximum and the minimum number of *active* processes during the temporal evolution of the system: if this difference is low, *z* should be chosen low as well. We thus define *z*, rounded to the nearest number, as:

10 $$  z = \frac {\max\limits_{v}(n^{v}_{act})-\min\limits_{v}(n^{v}_{act})}{2},  $$

where $n^{v}_{act}$ denotes the number of *active* processes in the *v*^*th*^ time window.

We eventually end up with a sequence of *z*+1 sub-models in the time interval [ 0,*T*], the first one being valid in $\left [0,t^{r}_{1}\right ]$ and the last one, in $\left [t^{r}_{z},T\right ]$.

Similarly to the global errors determined in “Model simplification by elimination of *always inactive* processes” section, we can also assess how the newly simplified models reproduce the dynamical behaviour of the original model in each time window $\left [t^{r}_{v-1},t^{r}_{v}\right ]$, by measuring the error:

11 $$  e^{v}_{h}=\frac{\int_{t^{r}_{v-1}}^{t^{r}_{v}}|y_{h}(t)-y_{h}^{r}(t)|dt}{\int_{t^{r}_{v-1}}^{t^{r}_{v}}|y_{h}(t)|dt}.  $$

We compute the error (11) between the original model and each sub-model, with or without propagating errors: in the first case, for each time window $\left [t^{r}_{v-1},t^{r}_{v}\right ]$ (*v*=1,…,*z*+1 with $t^{r}_{0}=t_{0}$ and $t^{r}_{z+1}=T$), the initial values of the *h* outputs of sub-model *SM*_*v*_ are equal to the final values at $t^{r}_{v-1}$ of sub-model *SM*_*v*−1_; in the second case, they are equal to the values of the original model at $t^{r}_{v-1}$.

### Global sensitivity analysis

Principal Process Analysis is applied to models with given parameter values and initial conditions. It may be questioned whether the uncertainty of their values influences the simplification of the model and thus, the analysis of the system dynamics. While we have shown PPA to be robust to variations of initial conditions in [21], the question remains open for parameter values.

To that aim, we perform global sensitivity analyses on the global relative errors between the original model and the reduced model (defined in Eq. (11)). Such an analysis consists in quantifying the parameter influence on the error, while varying the parameters simultaneously in given ranges. In contrast, in a local sensitivity analysis, parameters would vary one-at-a-time in the neighbourhood of their nominal value. First, we perform an analysis on each of the six errors defined for the six model outputs $ \left (e^{v}_{M_{P}},e^{v}_{M_{C}},e^{v}_{M_{B}},e_{P_{Tot}},e^{v}_{C_{Tot}},e^{v}_{B_{Tot}}\right)$. Then, in a more detailed analysis, we compute the global relative error for each state variable, according to Eq.(11) (with *y*_*h*_=*x*_*i*_,*i*=1,…,16); sensitivity analyses are also performed on each of these 16 errors. The method used is based on factorial design [22], analysis of variance (ANOVA) and principal component analysis (PCA) [23].

We first explore the parameter space using a factorial design. We vary *N*_*f*_=51 parameters of the model [17] (see “Model description” section). We choose *N*_*l*_=2 levels for each parameter *p*_*f*_ (or factor): $p^{-}_{f}= 0.8\: p_{f}$ and $p^{+}_{f}=1.2\: p_{f}$. A full factorial design, defined as all possible combinations of the parameter levels, would be necessary to estimate the main effects and interactions of all parameters. Such a full design corresponds to $N_{l}^{N_{f}}=2^{51}$ parameter combinations and would necessitate the same number of model simulations to compute the corresponding outputs, which are far too many. Thus we implement a fractional factorial design [24], which is a subset (fraction) of the full design of size $N_{j}<N_{l}^{N_{f}}$. The design is determined according to a given statistical model linking the error *e*_*h*_ to the parameters *p*_*f*_, for each time window $\left [t^{r}_{v-1},t^{r}_{v}\right ]$. We choose a second order linear model, which incorporates all main effects and two-way interactions as follows:

12 $$  e^{v}_{h,j}=\mu^{v}_{h}+\sum\limits_{f=1}^{N_{f}}\alpha^{v}_{h,f(j)}+\sum\limits_{f=1}^{N_{f}}\:\sum\limits_{k=1, k\neq f}^{N_{f}}\beta^{v}_{h,f(j)k(j)}+\epsilon^{v}_{h,j},  $$

where $e^{v}_{h,j}$ is the error computed according to Eq. (8) for output (or state variable) *h*, time window *v*, and parameter combination *j* (*j*=1,…,*N*_*j*_) of the fractional factorial design; $\mu ^{v}_{h}$ is the grand mean; $\alpha ^{v}_{h,f(j)}$ is the main effect of parameter *p*_*f*_ for parameter combination *j*; $\beta ^{v}_{h,f(j)k(j)}$ is the interaction effect between parameters *p*_*f*_ and *p*_*k*_ (*k*≠*f*) for parameter combination *j*; and $\epsilon ^{v}_{h,j}$ is the residual. Each main effect $\alpha ^{v}_{h,f(j)}$ can take two values, according to the level of parameter *p*_*f*_ in combination *j*: $\alpha ^{v}_{h,f^{+}}$ or $\alpha ^{v}_{h,f^{-}}$. Similarly, each two-way interaction effect can take four values: $\beta ^{v}_{h,f^{+}k^{+}},\: \beta ^{v}_{h,f^{+}k^{-}},\: \beta ^{v}_{h,f^{-}k^{+}},\: \beta ^{v}_{h,f^{-}k^{-}}$. The fractional factorial design determines the parameter combinations needed to estimate all main effects and two-way interactions. It is obtained using R package planor^2^ and consists of *N*_*j*_=2^12^ parameter combinations, yielding as many simulations. According to the sparsity-of-effects principle, a system is usually dominated by main effects and low order interactions, so neglecting third-order and higher interactions can still provide good estimates.

An ANOVA is then performed on these simulations, for each error *e*_*h*_. It consists in estimating the grand mean, main effects and interaction terms of model (12), using a least-square criterion to minimise the residuals. It is based on the following variance decomposition:

13 $$  \underbrace{\sum\limits_{j=1}^{N_{j}}\left(e^{v}_{h,j}-\overline{e^{v}_{h}}\right)^{2}}_{SS_{T}^{h,v}} = \underbrace{\sum\limits_{j=1}^{N_{j}}\left(\widehat{e^{v}_{h,j}}-\overline{e^{v}_{h}}\right)^{2}}_{SS_{M}^{h,v}} + \underbrace{\sum\limits_{j=1}^{N_{j}}\left(e^{v}_{h,j}-\widehat{e^{v}_{h,j}}\right)^{2}}_{SS_{r}^{h,v}=\sum_{j}(\epsilon^{v}_{h,j})^{2}},  $$

where $\overline {e^{v}_{h}}$ is the mean error computed over all *N*_*j*_ simulations of the fractional factorial design; and $\widehat {e^{v}_{h,j}}=\widehat {\mu ^{v}_{h}}+\sum _{f}\widehat {\alpha ^{v}_{h,f(j)}}+\sum _{f}\sum _{k\neq f}\widehat {\beta ^{v}_{h,f(j)k(j)}}$ ($\widehat {~}$ denoting an estimated value) is the error estimated from the linear model (12) for parameter combination *j*. The total sum of squares $SS_{T}^{h,v}$ is split into the sum of squares attributed to the model $SS_{M}^{h,v}$ and the residual sum of squares $SS_{r}^{h,v}$, the latter corresponding to the criterion that is minimised. In turn, $SS_{M}^{h,v}$ is split into sum of squares attributed to each main effect $\alpha ^{v}_{h,f}$ and two-way interaction term $\beta ^{v}_{h,fk}$, denoted respectively $SS_{f}^{h,v}$ and $SS_{fk}^{h,v}$. The total sensitivity index of parameter *p*_*f*_ is then defined as follows:

14 $$  tSI_{f}^{h,v}=\frac{SS_{f}^{h,v}+ \sum_{k\neq f} SS_{f,k}^{h,v}}{SS_{T}^{h,v}}.  $$

Noting that the variance of error $e^{v}_{h}$ computed over all *N*_*j*_ simulations of the fractional factorial design is $\sigma ^{2}_{e^{v}_{h}}=\frac {1}{N_{j}-1}SS_{T}^{h,v}$, the total sensitivity index $tSI_{f}^{h,v}$ represents the fraction of the variance explained by parameter *p*_*f*_. As an ANOVA requires a scalar variable, separate sensitivity analyses are performed for each scalar error $e^{v}_{h}$ and separate indices are computed for each error $e^{v}_{h}$. To compare the parameter influence on the different errors $e^{v}_{h}$, we use non normalised indices, obtained by multiplying each $tSI_{f}^{h,v}$ by the variance of the error:

15 $$  tSI_{f}^{h,v'}=\sigma^{2}_{e^{v}_{h}} tSI_{f}^{h,v}.  $$

To obtain synthetic sensitivity indices that represent the influence of each parameter on the errors for all 16 state variables, we decompose the error vector (*e*_*h*_:*h*=1…,16) by PCA (without normalisation). As a result, an inertia proportion *ω*_*c*_ can be attributed to each component *c* (a component is a linear combination of the 16 errors *e*_*h*_). It represents the variability among all simulations carried by this component. Only the *N*_*c*_ first components whose cumulated inertia add up to 95% or more are retained. Moreover, each simulation is given a *score* on each component, a scalar representing the projection of the simulation on the component. Then, for each component retained, an ANOVA is performed on the *scores* and total sensitivity indices $tSI^{c}_{f}$ are computed, as described in Eq. (14). Finally, a total generalised sensitivity index is calculated for each parameter *p*_*f*_ as the sum of the total sensitivity indices on each component, weighted by the inertia of the component:

16 $$  tGSI_{f}= \sum\limits_{c=1}^{N_{c}} w_{c} \: tSI^{c}_{f}.  $$

We use the multisensi R package^3^ for this analysis.

In what follows, we show how Principal Process Analysis can help with the analysis of complex biological models. We apply the approach to a model of the circadian clock developed in [17, 19], which we describe in the following section.

## Results

### Model description

**Fig. 3:**
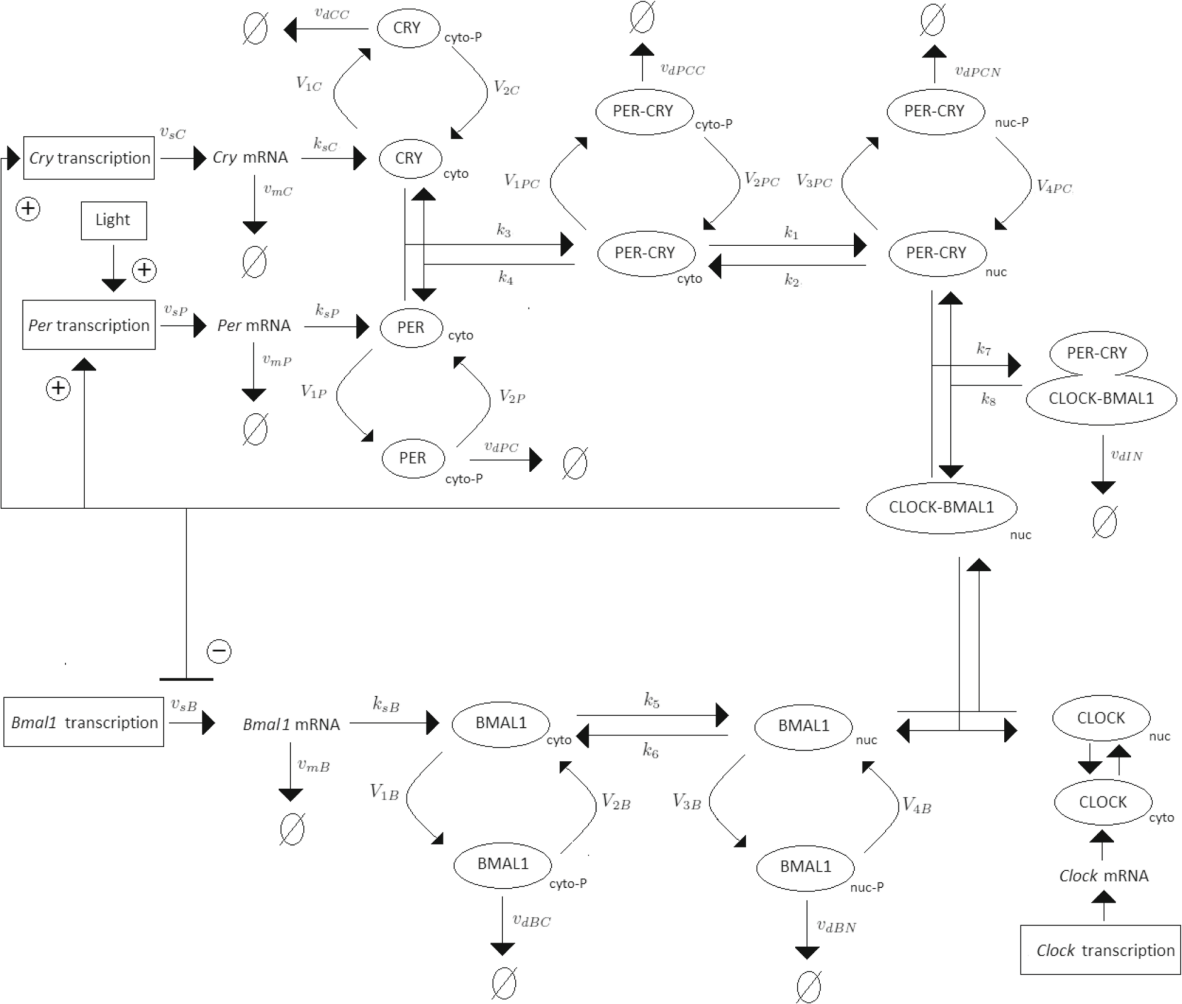
Schematic representation of the mammalian circadian clock. Light stimulates the transcription of gene *Per*. The complex CLOCK-BMAL1 inhibits the transcription of gene *Bmal1* and activates the transcription of genes *Cry* and *Per*. Notations: *∅*: degradation product; the different forms of a given protein are noted *cyto*: cytosolic form, *nuc*: nuclear form; *P*: phosphorylated form
- In the cytosol, the mRNAs can be either degraded or translated into proteins, which ones are subsequently phosphorylated (the process is reversible). Unphosphorylated proteins PER and CRY form the complex PER-CRY, which reversibly enters the nucleus. The nuclear and cytosolic forms of the complex can be phosphorylated. Likewise, protein BMAL1 is reversibly phosphorylated and reversibly enters the nucleus, but sole its unphosphorylated form makes a complex with protein CLOCK. Phosphorylated proteins and complexes in the nucleus or the cytosol are subject to degradation.
- In the nucleus, the complex CLOCK-BMAL1 activates the transcription of *Per* and *Cry* genes. Activation is stopped by binding of the PER-CRY complex to CLOCK-BMAL1, which indirectly inhibits *Per* and *Cry* transcription.
- The concentration of CLOCK protein is not a variable in the model because it is constitutively expressed at high levels and considered to be not limiting [17].

Periodic fluctuations of the environment subject living organisms to biological rhythms. The latter are endogenous by nature, but entrained by environmental variations. For instance, circadian rhythms are generated by a molecular clock within cells, which synchronizes daily physiological variations to the day-night alternance. The model we study here describes the circadian clock in mammals [17, 19]. In this model, the clock forms a complicated network of intertwined positive and negative feedback loops involving four clock genes: *Per*, *Cry*, *Bmal1*, and *Clock*. Their mRNA and protein produce sustained oscillations with a period of 24 hours. Light affects expression of gene *Per* at the transcriptional level: the first twelve hours of day light increase its transcription rate (up to 1.8 [ *μ*M/h]), while it is lowered in the next twelve hours of darkness (down to 1.5 [ *μ*M/h]). The system functions as follows (for the complete schema, see Fig. 3):

- Transcription of genes *Per*, *Cry* and *Bmal1* occurs in the nucleus. The newly synthesized mRNAs are exported to the cytosol.

The 16 model equations, 56 parameter and 16 initial condition values are shown in Appendix B. The model dynamics is difficult to analyse though, as the circadian clock involves numerous processes, including interlocked positive and negative feedback loops responsible for the oscillatory behaviour of the clock proteins. Reducing the original model around its core *active* processes can facilitate the model analysis, without changing significantly the original dynamics, in particular the sustained oscillations of the solutions.

### Principal Process Analysis of the circadian clock model

We apply Principal Process Analysis to identify major processes of the circadian clock model. To this end we decompose each ordinary differential equation in processes, as shown in Eq. (4) for BMAL1. Each process has a biological interpretation and corresponds to a regulatory mechanism or a biochemical reaction.

We then calculate the relative weight of each process using Eq. (5) and the threshold value *δ*=0.1.

**Fig. 4:**
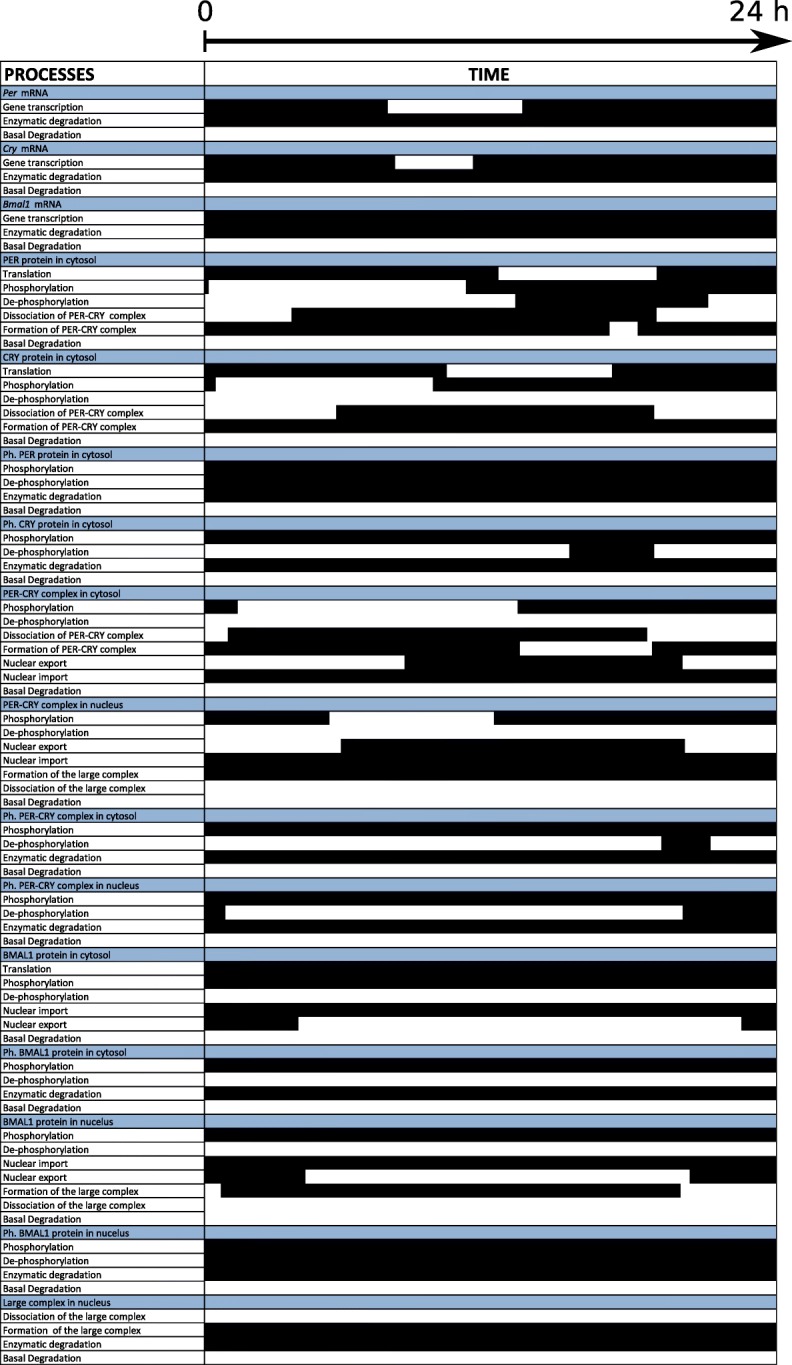
*Activity* of the 76 model processes during a 24-h period. Processes are listed in the first column (white background), ordered by variable (blue background). Their *activity* is depicted in the second column between 0 and 24 h: a horizontal black, resp. white, bar when the process is *active*, resp. *inactive*. Values for the *switching times* are given in Appendix B

We collect the *switching times* (values given in Appendix C) and then build a *Boolean Process Map* to visualize the *activity/inactivity* of each process, shown in Fig. 4. We simplify the model by neglecting 24 out of 76 processes, which are *always inactive* (32*%* of all processes). They correspond to mRNA and protein basal degradations; cytosolic dephosphorylations of CRY, BMAL1, and PER-CRY; PER-CRY-CLOCK-BMAL1 dissociation in the nucleus; and BMAL1 dephosphorylation in the nucleus. The list of neglected processes is shown in Appendix D.

We now determine the global relative errors between the original and the simplified model using Eq. (8) for all six outputs (see Table 1). The dynamics of the two models are compared in Fig. 7a. The simplified model preserves qualitatively the trend of the original solutions, as well as their sustained oscillations. The most noticeable difference concerns the peak of the total concentration of protein PER (*P*_*Tot*_), which corresponds also to the highest error in Table 1 (26.48%): the peak is lower with the reduced model, which also explains the delay between the original and the simplified solutions. Nevertheless the simplified model reproduces qualitatively the oscillatory behaviour of protein PER observed in the original model. The concentrations in the original and simplified models peak at almost the same time. These global relative errors do not call for an adjustment of the threshold value *δ*. In the next section, we proceed to the second step of the Principal Process Analysis.

**Table 1:** Global relative errors between the original and reduced models for the six outputs

Global relative error						
Output	*M*_*P*_	*M*_*C*_	*M*_*B*_	*P*_*Tot*_	*C*_*Tot*_	*B*_*Tot*_
Error	0.2499	0.2148	0.1535	0.2648	0.1326	0.2053

### Creation of sub-models

**Fig. 5:**
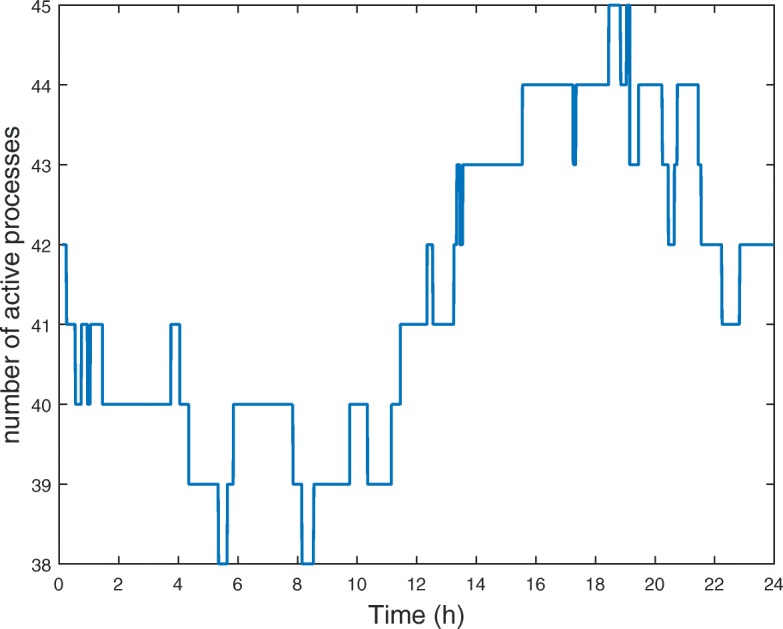
Evolution of the number of *active* processes as a function of time. The function increases or decreases at *switching times*, listed in Appendix B

**Fig. 6:**
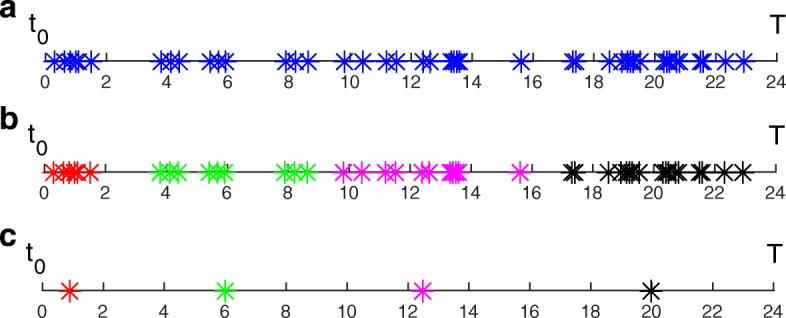
*Switching time* clustering. **a***switching times* *t*_*b*_, *b*=1,...,46 (also listed in Appendix B). **b** the four *switching time* clusters (red, green, pink, black) obtained by the k-means method. **c** the four reduced *switching times* ($t_{v}^{r}, v=1,...,4$), corresponding to the mean *switching time* within each cluster

The simplified model obtained above can be further reduced if we also neglect processes that are sometimes *inactive* during the system dynamics. Based on the *Boolean Process Map* and the collected *switching times*, we identify between 38 and 45 *active* processes along time (Fig. 5) and a total of 46 *switching times* (see Fig. 6a). Clustering the *switching times* into 4 clusters (Fig. 6b) allows us to generate the five sub-models described below. The number of clusters has been chosen according to Eq. (10).

**Fig. 7:**
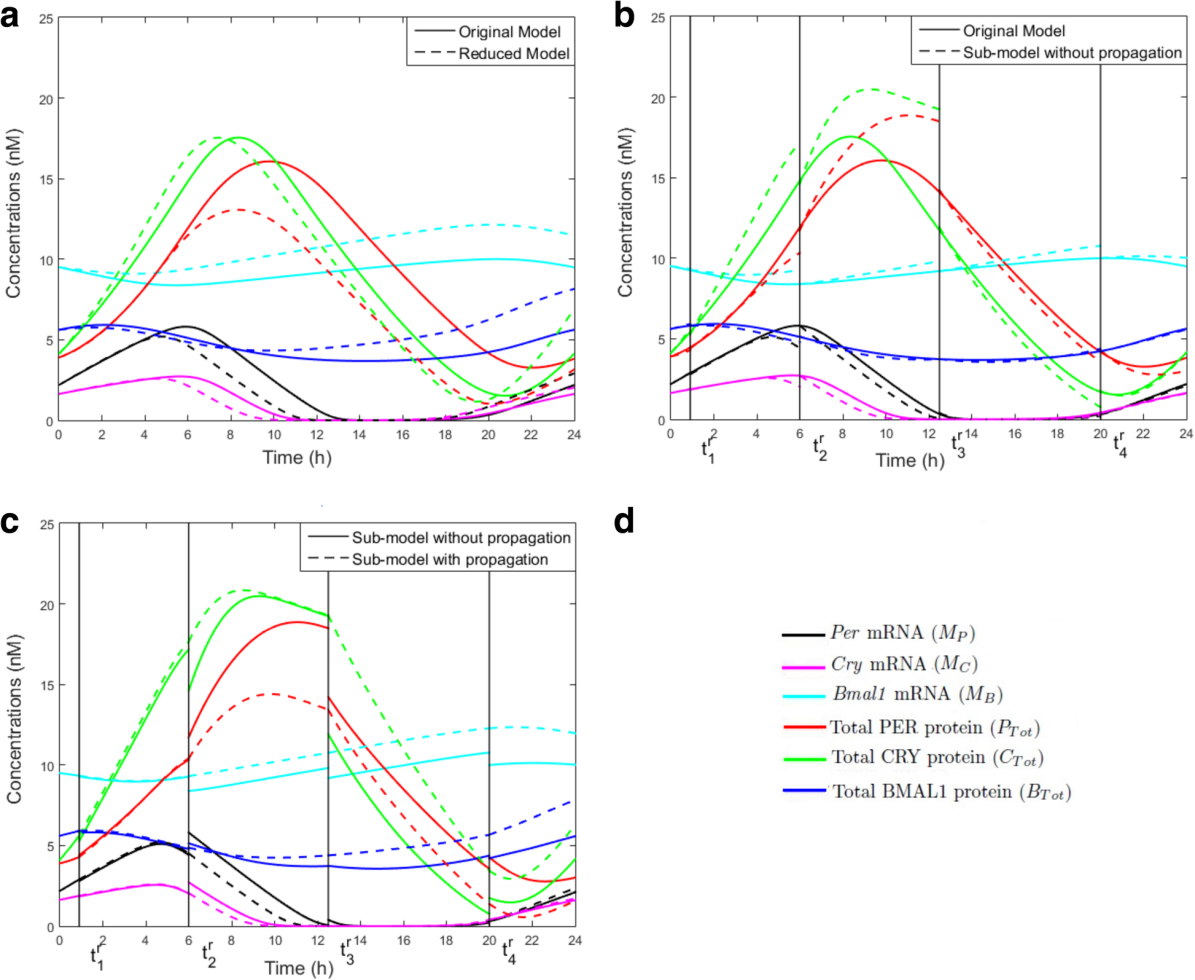
Model outputs along time for: **a** the original model (solid lines) and the reduced model (dashed lines); **b** the original model (solid lines) and the coupled sub-models without propagation errors (dashed lines); **c** the coupled sub-models, with (dashed lines) and without (solid lines) propagation errors. **d** The equations for the total concentration of protein PER (*P*_*Tot*_), CRY (*C*_*Tot*_) and BMAL1 (*B*_*Tot*_) are: *P*_*Tot*_=*P*_*C*_+*P*_*CP*_+*PC*_*C*_+*PC*_*N*_+*PC*_*CP*_+*PC*_*NP*_+*I*_*N*_, *C*_*Tot*_=*C*_*C*_+*C*_*CP*_+*PC*_*C*_+*PC*_*N*_+*PC*_*CP*_+*PC*_*NP*_+*I*_*N*_, *B*_*Tot*_=*B*_*C*_+*B*_*CP*_+*B*_*N*_+*B*_*NP*_+*I*_*N*_

- *SM*1, valid from $t_{0}^{r}=0$ to $t_{1}^{r}=0.9$ h: neglected processes for this model are *always inactive* (32% of the total). This model corresponds to the simplified model obtained in “Principal Process Analysis of the circadian clock model” section.
- *SM*2, from $t_{1}^{r}=0.9$ h to $t_{2}^{r}=6$ h: 46% of the processes are neglected. In addition to the *always inactive* listed in “Principal Process Analysis of the circadian clock model” section, we have the following *inactive* processes in this model: cytosolic dephosphorylation of PER, CRY, and PER-CRY; cytosolic dissociation of PER-CRY; nuclear dephosphorylation of PER-CRY; PER-CRY export from the nucleus; and formation of the large complex PER-CRY-CLOCK-BMAL1.
- *SM*3, from $t_{2}^{r}=6$ h to $t_{3}^{r}=12.5$ h, in which 50% of processes are neglected. In addition to the processes listed in “Principal Process Analysis of the circadian clock model” section, *inactive* processes are in this case: transcription of *Per* and *Cry* mRNAs; cytosolic phosphorylations and dephosphorylations of PER and CRY; cytosolic dephosphorylation of PER-CRY; nuclear phosphorylation and dephosphorylation of PER-CRY; and nuclear export of BMAL1.
- *SM*4, from $t_{3}^{r}=12.5$ h to $t_{4}^{r}=20$ h, which neglects 42% of processes. The processes include the processes listed in “Principal Process Analysis of the circadian clock model” section, as well as: PER and CRY translation; formation of the PER-CRY complex in the cytosol; PER-CRY dephosphorylation in the cytosol and the nucleus; and export of BMAL1 from the nucleus.
- *SM*5, from $t_{4}^{r}=20$ h to $t_{5}^{r}= 24$ h, in which 46% of the processes are neglected. With the processes listed in “Principal Process Analysis of the circadian clock model” section, other neglected processes are: cytosolic dephosphorylation of PER and CRY; PER-CRY dissociation in the cytosol; export of PER-CRY; PER-CRY dephosphorylation both in the cytosol and the nucleus; and PER-CRY-CLOCK-BMAL1 formation.

See also Appendix D for the list of neglected processes in each sub-model.

Table 2 gives the global relative errors (11) without propagation error, between the original model and the sub-models for the six outputs and for each time window. Figure 7b illustrates the six model outputs for the original model and the sub-models without propagation errors, while Fig. 7c compares the coupled sub-models with and without propagation error.The simplified models preserve the oscillatory behaviour of the total concentrations of PER, CRY, and BMAL1, albeit with some discrepancies in the amplitude of the oscillations. It is in the third time window that the approximated solution differs the most from the original one (Table 2). This is visible in Fig. 7b in the third time window where the total concentrations of PER and CRY form a much higher peak in the solution of the simplified model. Recall that this error is not an issue, since our objective is primarily the qualitative analysis of the model. It is sufficient that the remaining processes in the simplified model produce a dynamical behaviour qualitatively similar and relatively close to the original model. This shows their important contribution to the system dynamics.

**Table 2:** Global relative error between the original model and each sub-model without propagation error for the six outputs

Global relative error						
Output	*M*_*P*_	*M*_*C*_	*M*_*B*_	*P*_*Tot*_	*C*_*Tot*_	*B*_*Tot*_
Error SM1	0.0044	0.0044	0.0044	0.0208	0.0195	0.0073
Error SM2	0.0519	0.0434	0.0453	0.0397	0.1832	0.0402
Error SM3	0.2059	0.2951	0.0360	0.1427	0.2233	0.0356
Error SM4	0.0143	0.0377	0.0389	0.0678	0.1164	0.0210
Error SM5	0.0146	0.0032	0.0230	0.1150	0.0237	0.0053

**Fig. 8:**
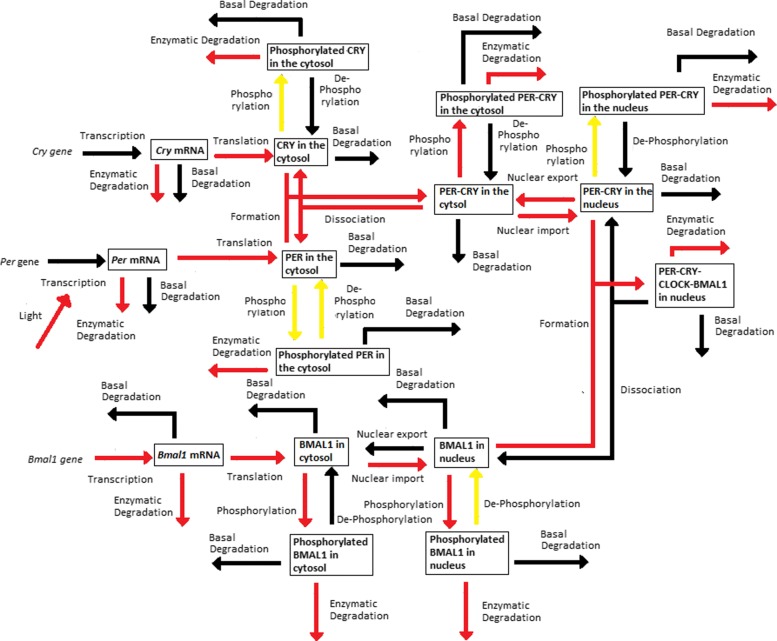
*Dynamical Process Map* for the third time window. Variables (boxes) and processes (arrows) are represented, as well as the process *activities*: *inactive* (black); *active* for all variables involved (red); *active* for some variables involved (yellow)

Applying a *Dynamical Process Map* to the third sub-model (Fig. 8; see also “Visualization of process *activities*” section) shows that the transcription of *Per* and *Cry* genes is *inactive* (black arrow) and that both PER and CRY phosphorylations in the cytosol and in the nucleus are not entirely *active* (they are not *active* for all the variables in which they are involved, yellow arrow). In the other time windows these processes are always entirely *active* (red arrows). This probably explains why we had an higher error in Table 2 for the variable *M*_*P*_, *M*_*C*_, *P*_*Tot*_ and *C*_*Tot*_ in SM3. The global sensitivity analysis, presented in the next Section, will confirm the validity of this assumption.

Since the dynamics of the coupled sub-models remain close to the original one, we can further analyse the behaviour of the network simplified to its core processes. We use the *Dynamical Process Maps* for the different sub-models (Appendix E), together with the process *activities* in Fig. 4 and the model outputs in Fig. 7. The simplified models preserve the three main interlocked feedback loops described in the original model, one positive and two negative loops. The functioning of these loops is directly affected by changes of process *activities*. Among the two negative feedback loops, which one is the main oscillator? One negative feedback loop involves the inhibition of *Bmal1* transcription by the nuclear form of BMAL1 associated to the protein CLOCK. If this mechanism is the main source of oscillations, we should observe wide changes in process *activities* controlling BMAL1 levels. The total concentration of the protein does not vary much in amplitude (Fig. 7). It mainly decreases in SM2 and SM3, when the concentration of PER-CRY is also high and forms a complex with CLOCK-BMAL1, which is subsequently degraded. This degradation process is *active* most of the time (Fig. 4 and Appendix E), but variations of the total BMAL1 concentration do not modify strongly the transcription of *Bmal1* mRNA, which remains always *active*. As well, the other processes of translation, phosphorylation and degradation for this variable almost never switch between *inactive* and *active* states over time (Fig. 4 and Appendix E). Overall, this suggests that the negative feedback loop involving CLOCK-BMAL1 is not the main oscillator. A similar conclusion was drawn for the original model in [17].

The other negative feedback loop inhibits *Per* and *Cry* transcription through the titration of CLOCK-BMAL1 by PER-CRY to form the inhibitory complex PER-CRY-CLOCK-BMAL1. The total concentration of BMAL1 peaks before that of PER and CRY, as can be seen in Fig. 7 for SM2 and SM3. When its concentration is maximal in SM1 and SM2, the nuclear form of the protein associated to the protein CLOCK stimulates the transcription of *Per* and *Cry* genes, in conditions where light has also a stimulatory effect on the transcription of these two genes. The processes of transcription and translation of *Per* and *Cry* are *active* in both models, as a result of which levels of PER and CRY raise to reach their maximal concentration in SM3. As can be seen from the process *activities* in Fig. 4 and the *Dynamical Process Maps* in Appendix E, conditions are favourable for the accumulation of high levels of complexes PER-CRY and CLOCK-BMAL1-PER-CRY in the nucleus. For instance, numerous processes decreasing PER, CRY and PER-CRY concentrations in the cytosol and the nucleus are *inactive*: their phosphorylation is reduced (the process is *inactive* for the dephosphorylated forms but still *active* for the phosphorylated ones), which limits their degradation, and the nuclear import of PER-CRY is always *active*. During the same period of time, the formation of the large complex CLOCK-BMAL1-PER-CRY, which is *active* for both CLOCK-BMAL1 and PER-CRY (Fig. 4 and Appendix E), suggests that the nuclear forms of PER-CRY and CLOCK-BMAL1 bind as soon as they accumulate in the nucleus. The large complex is immediately degraded since its degradation process is always *active* and its dissociation, *always inactive*.

In SM2 and SM3, the degradation of the large complex is not compensated for by other mechanisms allowing BMAL1 accumulation in the nucleus: the cytosolic form of the protein is *actively* phosphorylated and then degraded, while its dephosphorylation is *inactive*, which reduces the quantity of protein to be imported in the nucleus (see Fig. 4 and the *Dynamical Process Maps* in Appendix E). In this compartment, the absence of *active* dephosphorylation, together with the *active* protein phosphorylation, also contribute to decrease pools of CLOCK-BMAL1 complexes (Fig. 4, Appendix E). This halts transcription of *Per* and *Cry* mRNAs in SM3 (the processes are *inactive* and light is also switched off towards the end of SM3). This also affects the translation of PER and CRY, which becomes *inactive* in SM4. Altogether these observations suggest that the negative feedback loop inhibiting *Per* and *Cry* transcription via the complex CLOCK-BMAL1-PER-CRY is the main source of circadian oscillations. This is consistent with conclusions in [17], where a second oscillator based on the auto-inhibition of BMAL1 has been obtained for specific parameter values only. These results are also consistent with the observation of arrhythmic behaviours in mutant mice with double knock-out of the *Per* and *Cry* genes [25, 26].

The positive feedback loop activates *Per* and *Cry* transcription through a control of protein stability mediated by the phosphorylation processes. In the model, sole the phosphorylated forms of the proteins are degraded. We observed that the reversible phosphorylation reactions are often displaced in the forward sense, as dephosphorylation processes are often found *inactive*. In particular, they contribute to decrease the concentration of PER, CRY and PER-CRY, which also diminishes the concentration of the large complex CLOCK-BMAL1-PER-CRY and thus relieves the inhibition exerted by the complex on transcription of *Per* and *Cry* genes. Kinetic modelling of the circadian clock in *Drosophila* has shown the importance of this positive feedback loop for circadian rhythms [27].

### Influence of parameter values

**Fig. 9:**
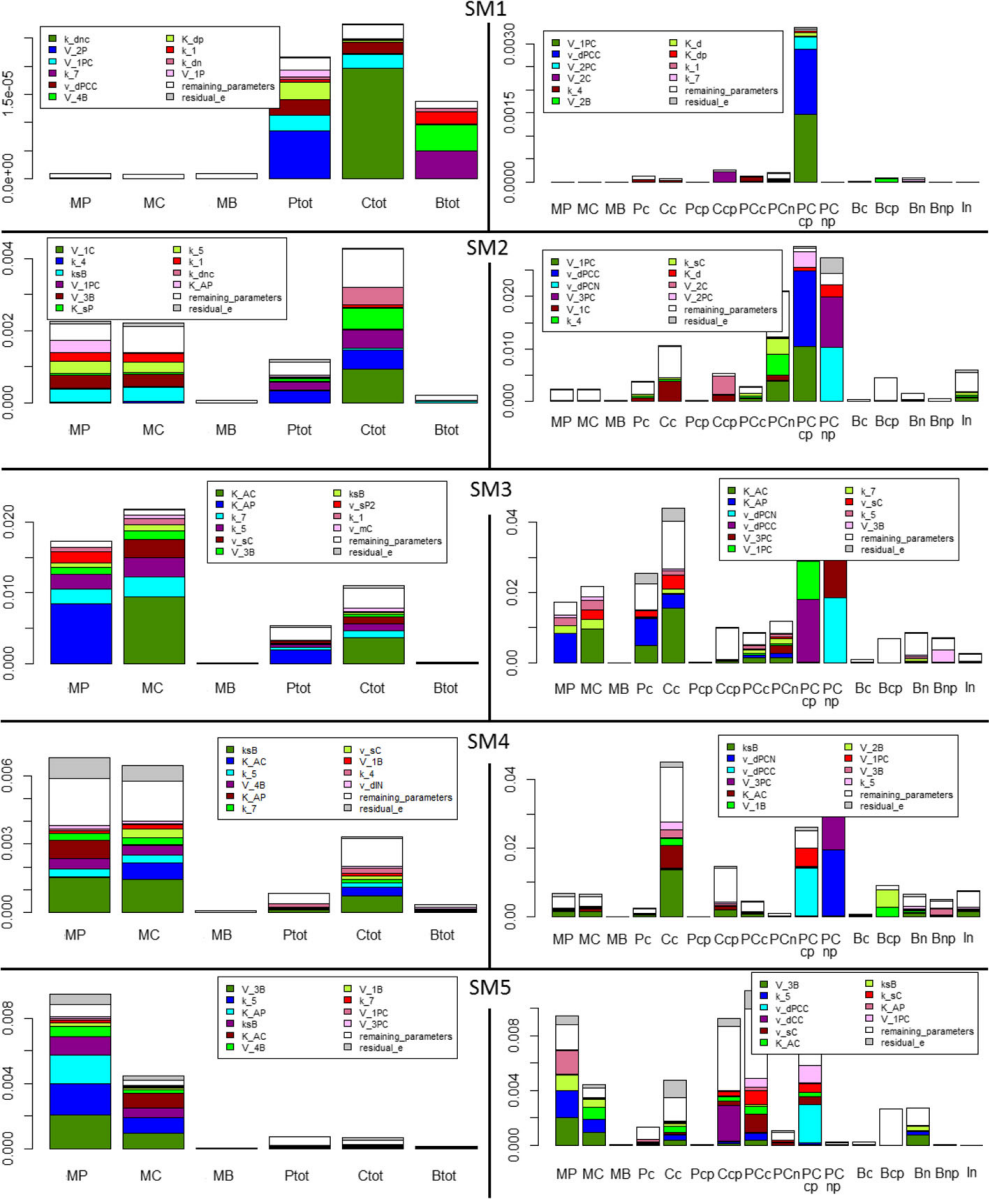
Global sensitivity analysis on the output (left column) or variable (right column) errors between the original model and the sub-models without propagation error for each time window (lines). Non-normalised total sensitivity indices are represented for each error (one bar per error) and for: (i) the 10 most influential parameters (color-coded); (ii) the remaining parameters (white). The residual is also represented (grey). For the biological meaning of the variables in the second column, see the equations in

In order to check the robustness of the five sub-models, we perform a global sensitivity analysis on the output errors for each time window $\left (e^{v}_{h}\right)$. We perform the analysis without propagating the errors because each sub-model is valid for a specific time window, independently from the other time windows. We vary 51 among the 56 parameters of the model: the Hill coefficients *m* and *n* are kept fixed because they represent the degree of cooperativity in gene repression/activation, while *k*_*stot*_, *v*_*stot*_, *V*_*phos*_ are function of other parameters (see Appendix B). We hence compute the non normalised total sensitivity indices for all parameters according to Eq. (15) (see Fig. 9, first column). Because the last three outputs (*P*_*Tot*_,*C*_*Tot*_,*B*_*Tot*_) are the sum of model variables that interact, some processes have no impact on these outputs and the information on the parameter influence is lost. We also perform the global sensitivity analysis on the 16 global relative errors between the original model and the sub-model variables without propagating errors (see Fig. 9, second column). The complex PER-CRY plays an important role in every time window: its variability is due mostly to its maximal phosphorylation velocity (*V*_1*PC*_) and its degradation parameter (*v*_*dPCC*_). In the third and fourth time window the other important variation is due to the CRY protein: in SM3 the variation is mostly due to the binding constants in the transcription of *Per* and *Cry* mRNAs (*K*_*AP*_ and *K*_*AC*_) and in SM4, to the maximal translation rate of BMAL1 (*k*_*sB*_) that stimulates *Per* and *Cry* mRNA transcription. In the last time window, lots of variables contribute to the system variation: the most important parameter for the variability of the outputs is the maximal velocity of BMAL1 phosphorylation in the nucleus (*V*_3*B*_).

To get a more global view of the model simplification, we calculate, for each parameter combination and for each time window, the average error (averaged over the 16 variables) between the original model and the sub-model variables as follows:

17 $$  \bar{e}^{v}=\frac{1}{16}\sum\limits_{i=1}^{16} e^{v}_{i}.  $$

**Fig. 10:**
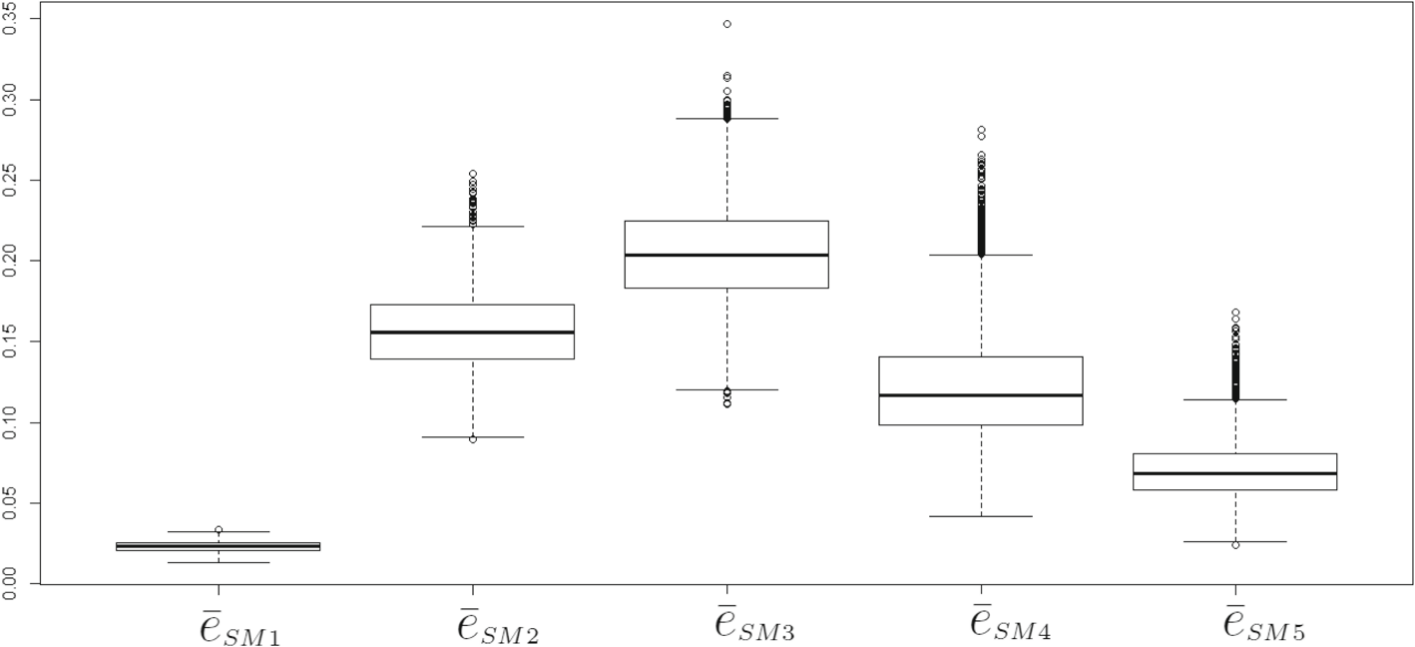
Average error between the original model and the sub-model variables calculated in each time window according to Eq. (17). Variability (box-plots) within each sub-model (or time window) is due to the various parameter combinations designed for the sensitivity analysis

Results are shown in Fig. 10. The variability is higher in the third and four sub-model, although the difference between the lower and upper quartiles is low in all sub-models.

**Fig. 11:**
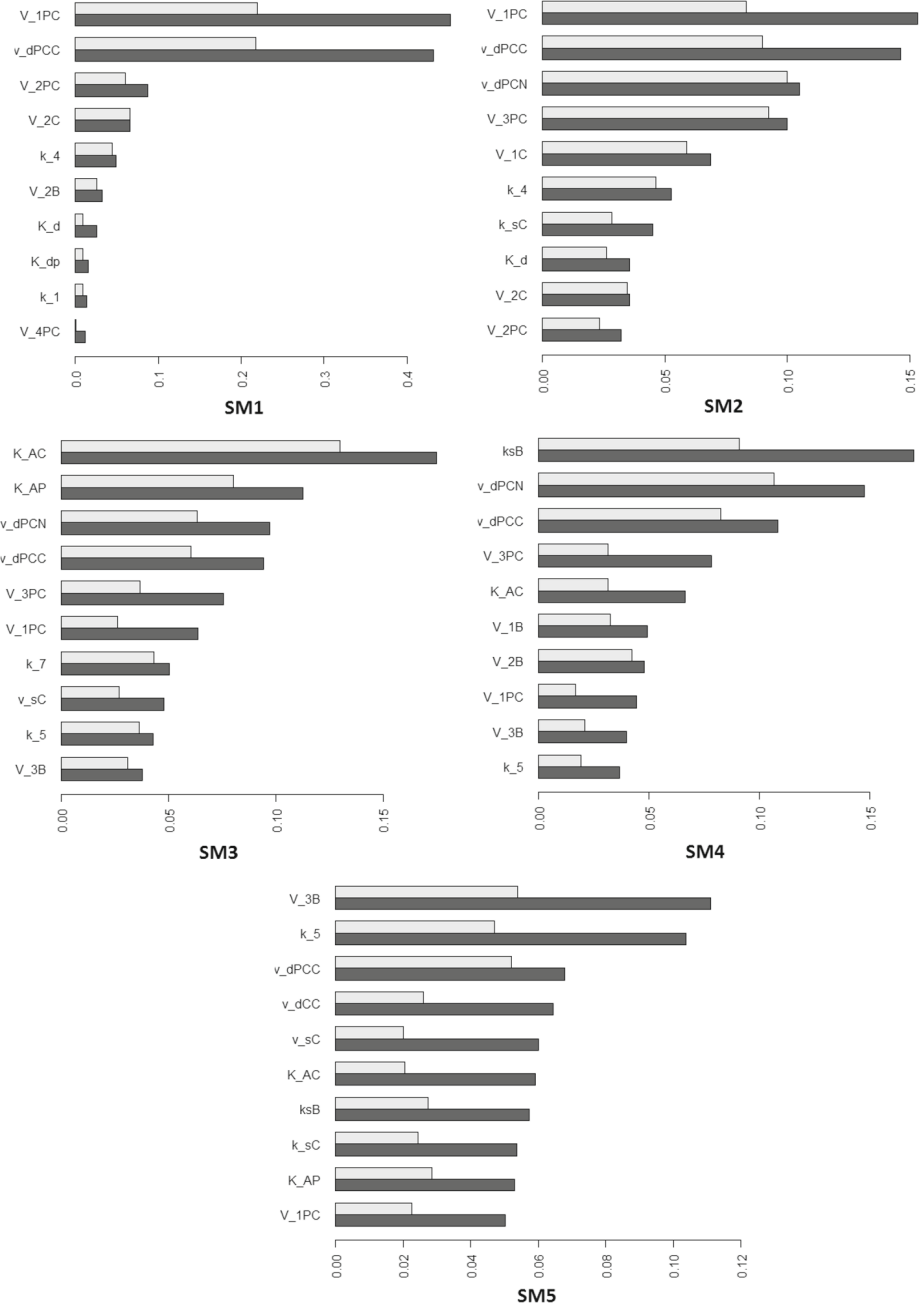
Generalised sensitivity indices (GSI) computed for each sub-model on the errors between the original model and the sub-model variables. The 10 most influential parameters on the errors are retained: main effect (grey bar) and total *GSI* (black bar)

Then, for each time-window, we compute the total generalised sensitivity indices according to Eq. (16), which represents the fraction of error variability explained by each parameter when parameter values vary. The results are shown in Fig. 11: we obtain similar results to the ones in Fig. 9 (column 2): in SM1 and SM2, the maximal phosphorylation velocity (*V*_1*PC*_) and degradation (*v*_*dPCC*_) of PER-CRY complex play the main role; in SM3, the binding constants of *Per* and *Cry* proteins (*K*_*AP*_ and *K*_*AC*_); in SM4, the translation of BMAL1 protein (*k*_*sB*_) and in SM5, the maximal phosphorylation velocity of BMAL1 protein in the nucleus (*V*_3*B*_). In order to check whether the error variations between the original model and the sub-models are due to parameters appearing in neglected processes, we determine the following ratio: $R^{v}_{h}=\frac {\sum \limits _{f\in \{ inactive~processes \}}tGSI_{f}^{h,v}}{\sum \limits _{f}tGSI_{f}^{h,v}}$. We only use the 10 most informative parameters, with higher *tGSI*, as they explain most variability. We choose a conservative option: if a parameter is neglected in an *inactive* process but still appears in other *active* processes, we still consider that it belongs to the neglected process parameters (worst case). Results are shown in Table 3. In most time windows, the variability is mainly due to parameters still contained in the reduced sub-models, i.e. the parameters of the *active* processes. In the third time-window, however, parameters appearing in neglected processes generate more than 50% of the variability. It is consistent with Fig. 7b: the peaks of the total concentration of PER and CRY are overestimated by the sub-model and some of the most important parameters that lead to the output variability for this time window are the translation rate of PER and CRY proteins, the maximal phosphorylation velocity of PER-CRY complex in the cytosol and nucleus (as it has been shown in Fig. 11). This confirms what we have supposed when applying the *Dynamical Process Map* to SM3 (see discussion about Fig. 8 at the end of “Creation of sub-models” section).

**Table 3:** Percentage of *tGSI* for parameters contained in *inactive* processes

% *tGSIinactive*					
*SM*	*SM*1	*SM*2	*SM*3	*SM*4	*SM*5
*R*_*h*_(*%*)	19.11	15.55	59.54	0	0

## Discussion

A challenging task when analysing the dynamics of biological networks is to understand the relation between the network behaviour and its numerous processes, the *activity* of which is switched on and off by regulatory mechanisms. Model reduction is one possible approach to deal with model complexity and help deciphering the design principle of these networks. However, the reduced models can be still too complex to study and this does not answer the question on the role of each individual process in the network behaviour. Ideally, one would like to identify the major processes, quantify and then understand their contribution to the system dynamics. Principal Process Analysis was developed with this objective in mind, and with the final goal of simplifying the original model in one or several sub-models around core *active* processes that are responsible for the dynamics of the original system. The dynamics of the core processes is much more tractable in the sub-models than in the original one. Questions remained open though concerning the scalability and robustness of this approach.

In this paper we tested the scalability of Principal Process Analysis by applying the approach on a model of high dimension, the mammalian circadian clock model, which incorporates numerous processes and complex interlocked feedback loops responsible for oscillatory behaviours. Simplification of the original system dynamics to as much as 50% of its processes in five coupled sub-models helped us relate the dynamics of the simplified models to the system components and their *active* interactions. We hence observed that the negative feedback loop controlling *Per* and *Cry* transcription through the formation of the large complex PER-CRY-CLOCK-BMAL1 is the main oscillator, in agreement with previous experimental and modelling studies [17, 25, 26]. Principal Process Analysis has been also applied with success to diverse biogeochemical and biochemical models in our group and elsewhere, see [14, 21, 28, 29]. These case studies and the present one exemplify the applicability and scalability of the approach to models of diverse nature and and complexity.

In this paper, the quantification of global errors allowed us to conclude that the simplified models reproduce well the behaviour of the original ones. Even in the case of the largest errors observed on the model output, did the simplified models preserve the oscillations of the clock proteins. Since Principal Process Analysis is based on the a priori knowledge of the model parameters, it was important to assess the robustness of the approach to uncertainties on these parameter values. Through a global sensitivity analysis, we studied the impact of of parameter values on the error between the original model and the simplified sub-models. Not only was the variation of the error small, but it was mostly due to parameters of the neglected processes. With this analysis, we proved the robustness of PPA to parameter uncertainty. In addition, we provided clues to identify and solve potential troubles related to the model simplification, in order to decrease errors between the original model and the simplified sub-models.

In a recent study, we also showed by other methods the robustness of PPA to initial conditions [21]. The latter were supposed to lie in rectangles contained in a region of the variable space varying by one order of magnitude in each coordinate. Under additional assumptions on the monotonicity of biological processes within rectangles, the maximal bound of process weights was computed, which allowed identifying *active* processes in each rectangle, similarly to “Principal Process Analysis (PPA)” section, for which weight is above the threshold value *δ*. Based on the behaviour of processes on the edges of the rectangles, it was then possible to determine the transitions between rectangles and deduce the evolution of process *activities* along the different transitions. The method has been applied on a small gene expression network containing a negative feedback loop [21]. The same principles could be applied to show the robustness of the model to larger variations of its parameters. In this case, the parameter space should be divided in rectangles in which the *activity/inactivity* of processes is studied. Such extension of the method is part of a future work.

In the current state of development, Principal Process Analysis is not a model reduction approach. For instance, the elimination of the *inactive* processes from the original model breaks down the mass conservation relations when eliminating a process in one equation that is considered *active* in others. As long as the purpose of PPA is to analyse the important processes in the original model, this is not an issue. Nevertheless, the approach could be extended so as to preserve mass conservation relations. In addition, simplified models with much smaller global relative errors could be obtained, so that the simplified sub-models represent more accurately the original model. We are currently studying a refinement of PPA by considering three different levels of *activities* (*inactive*, *active*, *fully active*), defined by two different thresholds in order to improve the quality of the model simplification and model analysis. Such improvements could bring PPA closer to a reduction method, since the simplified models become accurate representations of the original model.

## Conclusions

Mathematical models of biological systems have grown in complexity to include large numbers of processes. As a consequence, their contribution to the system dynamics becomes hardly tractable. The current manuscript contributes to this problem with the development of Principal Process Analysis. Provided the ODE model of the system is composed of a linear sum of terms describing each a process, the method enables the identification of the major processes contributing to the system dynamics and when they play a key role. Removing *inactive* processes allows restricting the dynamical analysis of the system to its core processes and facilitates the understanding of the system functioning. The conclusions derived with the method are robust to fluctuations in the parameter values. As such, Principal Process Analysis can be applied to any type of ODE models with the same form.

## Appendix A: Estimate of errors

We give in this appendix a rough estimate of the a priori error, based on bounds in the model and simple lemmas to compare the solutions of two differential equations. We refer to [18, Chapter 3], for the basic notions.

Consider the following ODE model of biological network, as given in Eq. (2):

18 $$  \dot{x}=f\left(x,p\right)  $$

Variable *x* is supposed to live in a bounded domain *D* of $\mathbb {R}^{n}$, and all functions in *f* are supposed to be smooth enough (at least *C*^2^) and Lipshitz on *D*. We denote by *L* the Lipschitz constant of *f*(*x*,*p*) on *D*. The decomposition into processes gives:

19 $$  \dot{x}_{i}=\sum_{j=1}^{n_{i}}f_{ij}\left(x,p\right) \;\; i=1,\ldots,n  $$

where *f*_*ij*_ represents the *j*^*th*^ process involved in the dynamical evolution of the *i*^*th*^ variable of the system over a period of time [ *t*_0_,*T*], and *n*_*i*_ is the number of processes for $\dot {x}_{i}$.

The weights are computed during [ *t*_0_,*T*]. For the sake of simplicity, we suppose that, for each variable, the weight of the first process and only this weight is lower than threshold *δ*:

20 $$  W_{i1}(t,p)=\frac{|f_{i1}(x(t),p)|}{\sum_{j}^{n_{i}}|f_{ij}(x(t),p)|} < \delta \;\; i=1,\ldots,n.  $$

It means that processes *f*_*i*1_,*i*=1,…,*n* are *inactive* during period [ *t*_0_,*T*], and thus eliminated from the system, giving the new simpler system (the new variable is denoted by *y* for simplicity):

21 $$  \dot{y}_{i}=\sum_{j=2}^{n_{i}}f_{ij}\left(y,p\right) \;\; i=1,\ldots,n.  $$

As variables are assumed to be bounded, vector *f*_1_=(*f*_11_,…,*f*_1*n*_)^*t*^ is such that:

22 $$ {}|f_{i1}(x,p)| \,=\, W_{i1}\;{\sum_{j=1}^{n_{i}}|f_{ij}(x(t),p)|} \leq \delta B_{i} \;\; \forall x \in D \;\; i\,=\,1,\ldots,n.  $$

where *B*_*i*_ is an upper bound for $\sum _{j=1}^{n_{i}}|f_{ij}(x(t),p)|$ obtained from the variable bounds on domain *D*. All *B*_*i*_ form vector *B*.

Therefore:

23 $$  ||f_{1}(x,p)|| \leq \delta ||B||  $$

If the initial conditions are the same (*x*(*t*_0_)=*y*(*t*_0_)), then Theorem 3.4 in [18], which is based on Gronwall’s Lemma, gives a bound between the two solutions *x* and *y*:

24 $$ ||x(t)- y(t)|| \leq \delta \; \frac{||B||}{L} \; { (e^{L(t-t_{0})}-1)} \; \forall t \in [t_{0}, T].  $$

The same proof applies when several *f*_*ij*_ are *inactive* for some variables. One just need to sum the errors in Eq. (22).

This gives a rough bound between the two solutions; this bound is theoretical and conservative, and is not used in the practical a posteriori computation of the error in our work. Nevertheless, it shows that the error is roughly proportional to the threshold *δ* used in the weight computations.

## Appendix B: Full mammalian model

### Model equations

Equations listed in [17, 19].mRNAs of *Per* gene

$$\begin{array}{lcl} \frac{dM_{P}}{dt} & = & v_{sP} \frac{B^{n}_{N}}{K^{n}_{AP}+B^{n}_{N}}- v_{mP} \frac{M_{P}}{K_{mP}+M_{P}}-k_{dmp} M_{P} ~~~~~~~~~~~~~~~~~~~~~~~~~~~~~~~~~~~~~~~ \\ \dot{x_{1}} & = & f_{1,1}+f_{1,2}+f_{1,3} \\ \end{array} $$

mRNAs of *Cry* gene

$$\begin{array}{lcl} \frac{dM_{C}}{dt} & = & v_{sC} \frac{B^{n}_{N}}{K^{n}_{AC}+B^{n}_{N}}- v_{mC} \frac{M_{C}}{K_{mC}+M_{C}}-k_{dmc} M_{C}~~~~~~~~~~~~~~~~~~~~~~~~~~~~~~~~~~~~~~~~~~~~~ \\ \dot{x_{2}} & = & f_{2,1}+f_{2,2}+f_{2,3} \\ \end{array} $$

mRNAs of *Bmal1* gene

$$\begin{array}{lcl} \frac{dM_{B}}{dt} & = & v_{sB} \frac{K^{n}_{IB}}{K^{n}_{IB}+B^{n}_{N}}- v_{mB} \frac{M_{B}}{K_{mB}+M_{B}}-k_{dmb} M_{B}~~~~~~~~~~~~~~~~~~~~~~~~~~~~~~~~~~~~~~~~~~~~~~~~~ \\ \dot{x_{3}} & = & f_{3,1}+f_{3,2}+f_{3,3} \\ \end{array} $$

Non-phosphorylated PER protein in the cytosol

$$\begin{array}{lcl} \frac{dP_{C}}{dt} & = & k_{sP} M_{P} - V_{1P} \frac{P_{C}}{K_{P}+P_{C}}+ V_{2P} \frac{P_{CP}}{K_{dP}+P_{CP}} \\&+& k_{4} PC_{C} -k_{3} P_{C} C_{C} - k_{dn} P_{C} ~~~~~~~~~~~~~~~~~~~~~~~~~~~~~~~~~~~~~~~~~~~~~~~~\\ \dot{x_{4}} & = & f_{4,1}+f_{4,2}+f_{4,3}+f_{4,4}+f_{4,5}+f_{4,6} \\ \end{array} $$

Non-phosphorylated CRY protein in the cytosol

$$\begin{array}{lcl} \frac{dC_{C}}{dt} &=& k_{sC} M_{C} - V_{1C} \frac{C_{C}}{K_{P}+C_{C}}+ V_{2C} \frac{C_{CP}}{K_{dP}+C_{CP}}\\ &+& k_{4} PC_{C} -k_{3} P_{C} C_{C} - k_{dnc} C_{C} ~~~~~~~~~~~~~~~~~~~~~~~~~~~~~~~~~~~~~~~~~~~~~~~~\\ \dot{x_{5}} & = & f_{5,1}+f_{5,2}+f_{5,3}+f_{5,4}+f_{5,5}+f_{5,6} \\ \end{array} $$

Phosphorylated PER protein in the cytosol

$${}\begin{array}{lcl} \frac{dP_{CP}}{dt} & = &V_{1P} \frac{P_{C}}{K_{P}+P_{C}}- V_{2P} \frac{P_{CP}}{K_{dP}+P_{CP}}- v_{dPC} \frac{P_{CP}}{K_{d}+P_{CP}} - k_{dn} P_{CP}~~~~~~~~~~~~~~~~~~~~~~~~~~~~~~~~~~~~~~~~~~~~~~~~ \\ \dot{x_{6}} & = & f_{6,1}+f_{6,2}+f_{6,3}+f_{6,4}\\ \end{array} $$

Phosphorylated CRY protein in the cytosol

$$\begin{array}{lcl} {}\frac{dC_{CP}}{dt} & = &\!\! V_{1C} \frac{C_{C}}{K_{P}+C_{C}}\,-\, V_{2C} \frac{C_{CP}}{K_{dP}+C_{CP}}\,-\, v_{dCC} \frac{C_{CP}}{K_{d}+C_{CP}} \,-\, k_{dn} C_{CP}~~~~~~~~~~~~~~~~~~~~~~~~~~~~~~~~~~~~~~~~~~~~~~~~ \\ \dot{x_{7}} & = & f_{7,1}+f_{7,2}+f_{7,3}+f_{7,4}\\ \end{array} $$

Non-phosphorylated PER-CRY complex in the cytosol

$$\begin{array}{lcl} \frac{dPC_{C}}{dt} & = &-V_{1PC} \frac{PC_{C}}{K_{P}+PC_{C}}+ V_{2PC} \frac{PC_{CP}}{K_{dP}+PC_{CP}}- k_{4} PC_{C}\\ & &+k_{3} P_{C} C_{C} + k_{2} PC_{N}- k_{1} PC_{C} - k_{dn} PC_{C}~~~~~~~~~~~~~~~~~~~~~~~~~~~~~~~~~~~~~~~~~~~~~~~~\\ \dot{x_{8}} & = & f_{8,1}+f_{8,2}+f_{8,3}+f_{8,4}+f_{8,5}+f_{8,6}+f_{8,7}\\ \end{array} $$

Non-phosphorylated PER-CRY complex in the nucleus

$$\begin{array}{lcl} {}\frac{dPC_{N}}{dt} & = &\!-V_{3PC} \frac{PC_{N}}{K_{P}+PC_{N}}\,+\, V_{4PC} \frac{PC_{NP}}{K_{dP}+PC_{NP}}\,-\, k_{2} PC_{N}\,+\,k_{1}\! PC_{C} \\ & &\!-k_{7} B_{N} PC_{N} + k_{8} I_{n} -k_{dn} PC_{N} ~~~~~~~~~~~~~~~~~~~~~~~~~~~~~~~~~~~~~~~~~~~~~~~~\\ {}\dot{x_{9}} & = & f_{9,1}+f_{9,2}+f_{9,3}+f_{9,4}+f_{9,5}+f_{9,6}+f_{9,7}\\ \end{array} $$

Phosphorylated PER-CRY complex in the cytosol

$$ {}\begin{array}{lcl} \frac{dPC_{CP}}{dt} \!\!& = &\!\!V_{1PC} \frac{PC_{C}}{K_{P}+PC_{C}}\,-\, V_{2PC}\! \frac{PC_{CP}}{K_{dP}+PC_{CP}}\,-\, v_{dPCC}\ \frac{PC_{CP}}{K_{d}+PC_{CP}}\,-\, k_{dn} PC_{CP}\\ \dot{x_{10}} \!& = &\! f_{10,1}+f_{10,2}+f_{10,3}+f_{10,4}\\ \end{array}  $$

Phosphorylated PER-CRY complex in the nucleus

$$ {} \begin{array}{lcl} \frac{dPC_{NP}}{dt} \!\!& = &\!\!\!V_{3PC}\! \frac{PC_{N}}{K_{P}+PC_{N}}\,-\, V_{4PC}\! \frac{PC_{NP}}{K_{dP}+PC_{NP}}\,-\, v_{dPCN} \frac{PC_{NP}}{K_{d}\,+\,PC_{NP}}\,-\, k_{dn} PC_{NP}~~~~~~~~~~~~~~~~~~~~~~~~~~~~~~~~~~~~~~~~~~~~~~~~\\ \dot{x_{11}} \!& = &\! f_{11,1}+f_{11,2}+f_{11,3}+f_{11,4}\\ \end{array}  $$

Non-phosphorylated BMAL1 protein in the cytosol

$$ {} \begin{array}{lcl} \frac{dB_{C}}{dt} & = & k_{sB} M_{B} \,-\, V_{1B} \frac{B_{C}}{K_{P}+B_{C}}\,+\, V_{2B} \frac{B_{CP}}{K_{dP}+B_{CP}} -k_{5} B_{C} +k_{6} B_{N} - k_{dn} B_{C} ~~~~~~~~~~~~~~~~~~~~~~~~~~~~~~~~~~~~~~~~~~~~~~~~\\ \dot{x_{12}} & = & f_{12,1}+f_{12,2}+f_{12,3}+f_{12,4}+f_{12,5}+f_{12,6} \\ \end{array}  $$

Phosphorylated BMAL1 protein in the cytosol

$${}\begin{array}{lcl} \frac{dB_{CP}}{dt} & = & V_{1B} \frac{B_{C}}{K_{P}+B_{C}}- V_{2B} \frac{B_{CP}}{K_{dP}+B_{CP}}- v_{dBC} \frac{B_{CP}}{K_{d}+B_{CP}} -k_{dn} B_{CP} ~~~~~~~~~~~~~~~~~~~~~~~~~~~~~~~~~~~~~~~~~~~~~~~~\\ \dot{x_{13}} & = & f_{13,1}+f_{13,2}+f_{13,3}+f_{13,4} \\ \end{array} $$

Non-phosphorylated BMAL1 protein in the nucleus

$${}\begin{array}{lcl} \frac{dB_{N}}{dt} \!\!\!& = &\! \,-\,V_{3B} \frac{B_{N}}{K_{P}+B_{N}}\,+\,\! V_{4B} \frac{B_{NP}}{K_{dP}+B_{NP}}\,+\, k_{5} B_{C} \,-\, k_{6} B_{N} \,-\,k_{7} B_{N} PC_{N} \\ & &+k_{8} I_{N} -k_{dn} B_{N} ~~~~~~~~~~~~~~~~~~~~~~~~~~~~~~~~~~~~~~~~~~~~~~~~\\ \dot{x_{14}} & = & f_{14,1}+f_{14,2}+f_{14,3}+f_{14,4}+f_{14,5}+f_{14,6}+f_{14,7} \\ \end{array} $$

Phosphorylated BMAL1 protein in the nucleus

$${}\begin{array}{lcl} \frac{dB_{NP}}{dt} & = & V_{3B} \frac{B_{N}}{K_{P}+B_{N}}\,-\, V_{4B} \frac{B_{NP}}{K_{dP}+B_{NP}} - v_{dBN} \frac{B_{NP}}{K_{d}+B_{NP}} -k_{dn} B_{NP}~~~~~~~~~~~~~~~~~~~~~~~~~~~~~~~~~~~~~~~~~~~~~~~~\\ \dot{x_{15}} & = & f_{15,1}+f_{15,2}+f_{15,3}+f_{15,4}\\ \end{array} $$

Inactive complex between PER-CRY and CLOCK-BMAL1 in the nucleus

$$\begin{array}{lcl} \frac{dI_{N}}{dt} & = & -k_{8} I_{N} + k_{7} B_{N} PC_{N} - v_{dIN} \frac{I_{N}}{K_{d} + I_{N}} - k_{dn} I_{N}~~~~~~~~~~~~~~~~~~~~~~~~~~~~~~~~~~~~~~~~~~~~~~~~\\ \dot{x_{16}} & = & f_{16,1}+f_{16,2}+f_{16,3}+f_{16,4}\\ \end{array} $$

### Model parameters

Parameters listed in [17, p.546]: Set 1.

*k*_1_(*h*^−1^)=0.4, *k*_2_(*h*^−1^)=0.2, *k*_3_(*nM*^−1^*h*^−1^)=0.4, *k*_4_(*h*^−1^)=0.2, *k*_5_(*h*^−1^)=0.4, *k*_6_(*h*^−1^)=0.2, *k*_7_(*nM*^−1^*h*^−1^)=0.5, *k*_8_(*h*^−1^)=0.1, *K*_*AP*_(*nM*)=0.7, *K*_*AC*_(*nM*)=0.6, *K*_*IB*_(*nM*)=2.2, *k*_*dmb*_(*h*^−1^)=0.01, *k*_*dmc*_(*h*^−1^)=0.01, *k*_*dmp*_(*h*^−1^)=0.01, *k*_*dnc*_(*h*^−1^)=0.12, *k*_*dn*_(*h*^−1^)=0.01, *K*_*d*_(*nM*)=0.3, *K*_*dp*_(*nM*)=0.1, *K*_*p*_(*nM*)=0.1, *K*_*mB*_(*nM*)=0.4, *K*_*mC*_(*nM*)=0.4, *K*_*mP*_(*nM*)=0.31, *k*_*stot*_(*h*^−1^)=1.0, *k*_*sB*_(*h*^−1^)=0.12*k*_*stot*_, *k*_*sC*_(*h*^−1^)=1.6*k*_*stot*_, *k*_*sP*_(*h*^−1^)=0.6*k*_*stot*_, *n*=4, *m*=2, *V*_*phos*_(*nMh*^−1^)=0.4, *V*_1*B*_(*nMh*^−1^)=0.5, *V*_1*C*_(*nMh*^−1^) = 0.6, *V*_1*P*_(*nMh*^−1^) = *V*_*phos*_,*V*_1*PC*_(*nMh*^−1^)=*V*_*phos*_, *V*_2*B*_(*nMh*^−1^)=0.1, *V*_2*C*_(*nMh*^−1^)=0.1, *V*_2*P*_(*nMh*^−1^)=0.3, *V*_2*PC*_(*nMh*^−1^)=0.1, *V*_3*B*_(*nMh*^−1^)=0.5, *V*_3*PC*_(*nMh*^−1^)=*V*_*phos*_, *V*_4*B*_(*nMh*^−1^)=0.2, *V*_4*PC*_(*nMh*^−1^)=0.1, *v*_*dBC*_(*nMh*^−1^)=0.5, *v*_*dBN*_(*nMh*^−1^)=0.6*v*_*dCC*_(*nMh*^−1^)=0.7, *v*_*dIN*_(*nMh*^−1^)=0.8, *v*_*dIN*_(*nMh*^−1^)=0.8, *v*_*dPC*_(*nMh*^−1^)=0.7, *v*_*dPCC*_(*nMh*^−1^) = 0.7,*v*_*dPCN*_(*nMh*^−1^) = 0.7,*v*_*mB*_(*nMh*^−1^) =0.8, *v*_*mC*_(*nMh*^−1^)=1.0, *v*_*mP*_(*nMh*^−1^)=1.1, *vstot*(*nMh*^−1^)=1.0, *v*_*sB*_(*nMh*^−1^)=*v*_*stot*_, *v*_*sB*_(*nMh*^−1^)=*v*_*stot*_, *v*_*sC*_(*nMh*^−1^)=1.1*v*_*stot*_, *v*_*sP*_(*nMh*^−1^)=1.5*v*_*stot*_

**Table 4:** *Switching times* (s.t), their values (v.) in [h] and associate reduced (cluster) *switching times*$\left (t^{r}_{1},t^{r}_{2},t^{r}_{3},t^{r}_{4}\right)$ (s.t.c.): $t_{1}^{r}$ is associated to the cluster of $t_{1}- t_{6}, t^{r}_{2}$ to $t_{7} - t_{15}, t^{r}_{3}$ to *t*_16_−*t*_26_, and $t^{r}_{4}$ to *t*_27_−*t*_46_

s.t.	v.	s.t.c.	s.t.	v.	s.t.c.	s.t.	v.	s.t.c.	s.t.	v.	s.t.c.
*t*_0_	0		*t*_12_	5.9	6	*t*_24_	13.5		*t*_36_	19.5	20
*t*_1_	0.3		*t*_13_	7.9		*t*_25_	13.6		*t*_37_	20.3	
*t*_2_	0.6		*t*_14_	8.2		*t*_26_	15.6		*t*_38_	20.4	
*t*_3_	0.8	0.9	*t*_15_	8.6		*t*_27_	17.3		*t*_39_	20.45	
*t*_4_	1		*t*_16_	9.8		*t*_28_	17.4		*t*_40_	20.5	
*t*_5_	1.1		*t*_17_	10.4		*t*_29_	18.5		*t*_41_	20.7	
*t*_6_	1.5		*t*_18_	11.2		*t*_30_	18.9		*t*_42_	20.8	
*t*_7_	3.8		*t*_19_	11.5		*t*_31_	19.1		*t*_43_	21.5	
*t*_8_	4.1		*t*_20_	12.4	12.5	*t*_32_	19.2		*t*_44_	21.6	
*t*_9_	4.4		*t*_21_	12.6		*t*_33_	19.25		*t*_45_	22.3	
*t*_10_	5.4		*t*_22_	13.3		*t*_34_	19.3		*t*_46_	22.9	
*t*_11_	5.7		*t*_23_	13.4		*t*_35_	19.35		*T*	24	

### Initial conditions

The unit of the initial conditions is *nM*.

*M*_*P*_(0)=2.188*M*_*C*_(0)=1.633,*M*_*B*_(0)=9.498,*P*_*C*_(0)=2.008,*C*_*C*_(0)=1.884,*P*_*CP*_(0)=0.129,*C*_*CP*_(0)=0.473, *PC*_*C*_(0)=1.228,*PC*_*N*_(0)=0.177,*PC*_*CP*_(0)=0.203, *PC*_*NP*_(0)= 0.101,*B*_*C*_(0) =2.523,*B*_*CP*_(0)=0.929,*B*_*N*_(0)=1.787,*B*_*NP*_(0)=0.318,*I*_*N*_(0)=0.051

## Appendix C: Switching times

See Table 4.

## Appendix D: Neglected processes

### First reduced model

Neglected processes are: *f*_1,3_, *f*_2,3_, *f*_3,3_, *f*_4,6_, *f*_5,3_, *f*_5,6_, *f*_6,4_, *f*_7,4_, *f*_8,2_, *f*_8,7_, *f*_9,6_, *f*_9,7_, *f*_10,4_, *f*_11,4_, *f*_12,3_, *f*_12,6_, *f*_13,2_, *f*_13,4_, *f*_14,2_, *f*_14,6_, *f*_14,7_, *f*_15,4_, *f*_16,1_, *f*_16,4_.

### Second reduced model: sub-models

Neglected processes in SM1 are: *f*_1,3_, *f*_2,3_, *f*_3,3_, *f*_4,6_, *f*_5,3_, *f*_5,6_, *f*_6,4_, *f*_7,4_, *f*_8,2_, *f*_8,7_, *f*_9,6_, *f*_9,7_, *f*_10,4_, *f*_11,4_, *f*_12,3_, *f*_12,6_, *f*_13,2_, *f*_13,4_, *f*_14,2_, *f*_14,6_, *f*_14,7_, *f*_15,4_, *f*_16,1_, *f*_16,4_.

In SM2, we supposed that processes switching state from *t*_1_=0.33 until *t*_6_=1.5 change simultaneously at time $t_{1}^{r}=0.9$. Deleted processes are common to those removed in SM1, as well as: *f*_4,3_, *f*_4,4_, *f*_5,4_, *f*_7,2_, *f*_8,3_,*f*_8,5_, *f*_9,2_, *f*_9,3_, *f*_10,2_, *f*_14,5_.

In SM3, we supposed that processes switching state from *t*_7_=3.8 until *t*_6_=1.5 change simultaneously at time $t_{2}^{r}=6$. Deleted processes are common to those removed in SM1, as well as: *f*_1,1_, *f*_2,1_, *f*_4,2_, *f*_4,3_, *f*_5,2_, *f*_7,2_, *f*_8,1_, *f*_9,1_, *f*_9,2_, *f*_10,2_, *f*_11,2_, *f*_12,5_, *f*_14,4_.

In SM4, we supposed that processes switching state from *t*_16_=9.8 until *t*_26_=15.6 change simultaneously at time $t_{3}^{r}=12.5$. Deleted processes are common to those removed in SM1, as well as: *f*_4,1_, *f*_5,1_, *f*_8,4_, *f*_9,2_, *f*_10,2_, *f*_11,2_,*f*_12,5_, *f*_14,4_.

In SM5, we supposed that processes switching state from *t*_27_=17.3 until *t*_46_=22.9 change simultaneously at time $t_{4}^{r}=20.0$. Deleted processes are common to those removed in SM1, as well as: *f*_4,3_, *f*_4,4_, *f*_5,4_, *f*_7,2_, *f*_8,3_, *f*_8,5_, *f*_9,2_, *f*_9,3_, *f*_10,2_, *f*_14,5_.

## Appendix E: *Dynamical Process Maps*

**Fig. 12:**
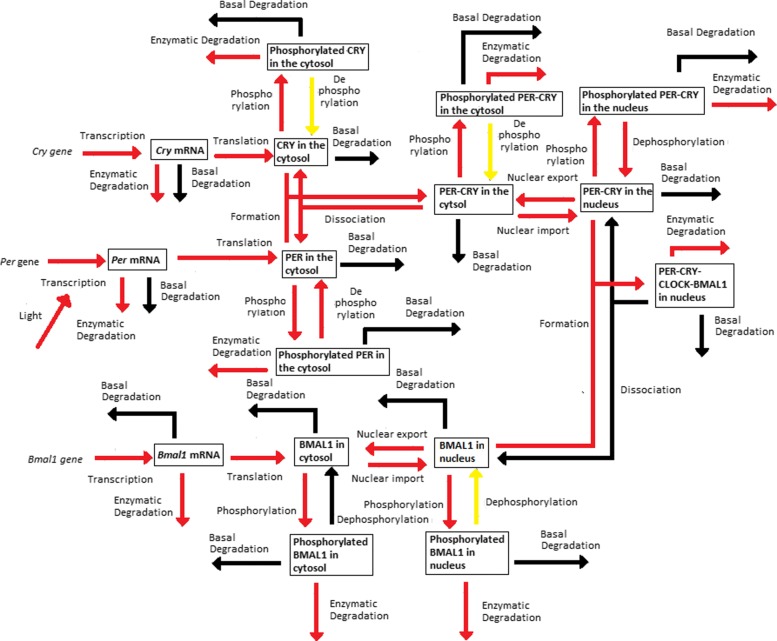
*Dynamical Process Map* of SM1

**Fig. 13:**
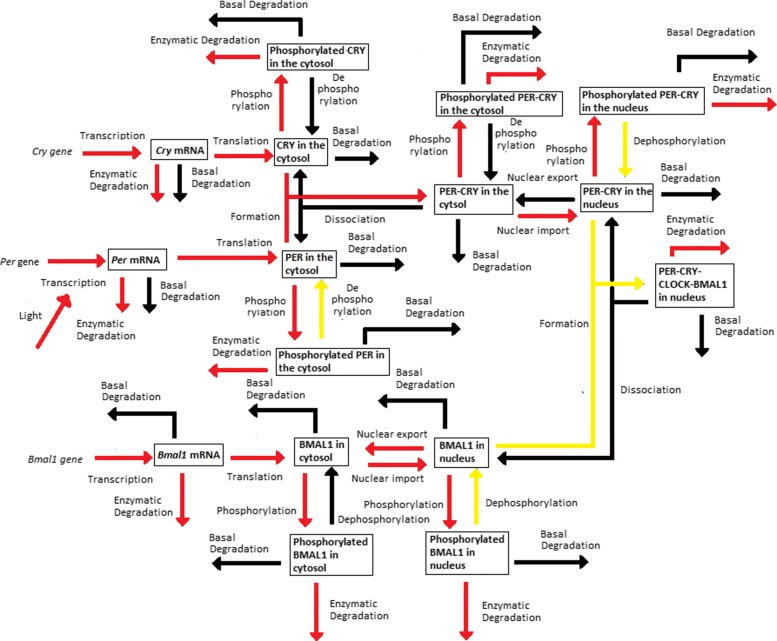
*Dynamical Process Map* of SM2

**Fig. 14:**
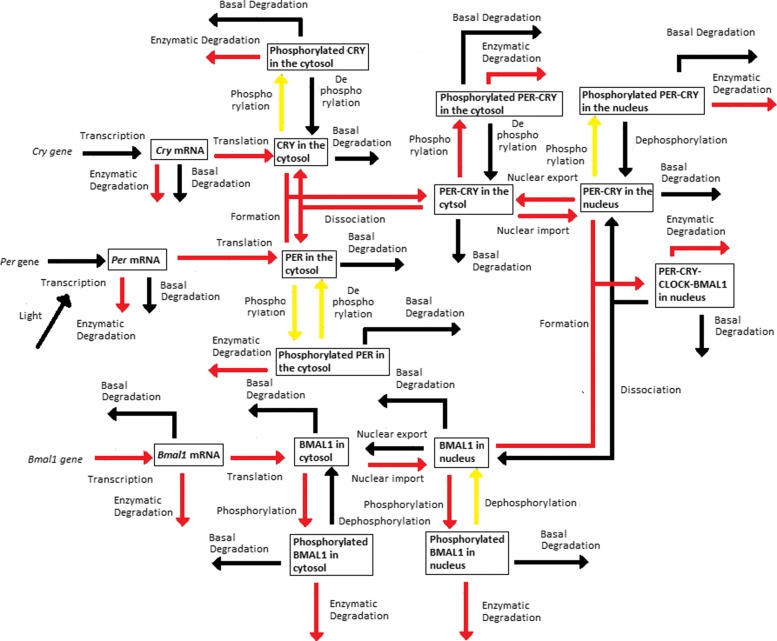
*Dynamical Process Map* of SM3

**Fig. 15:**
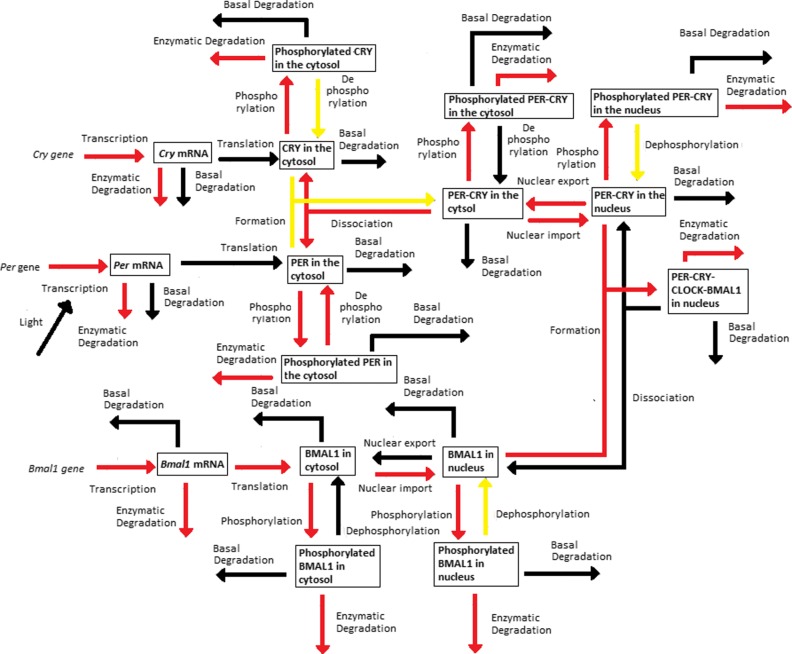
*Dynamical Process Map* of SM4

**Fig. 16:**
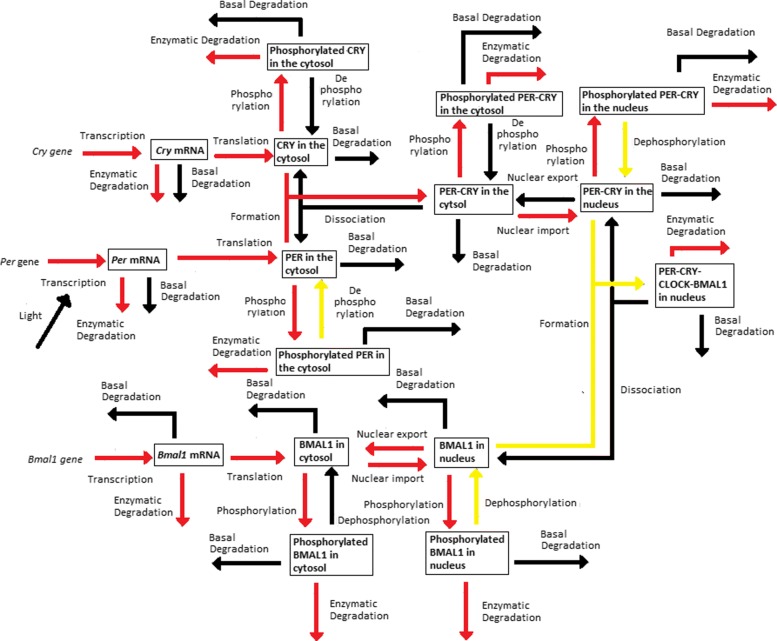
*Dynamical Process Map* of SM5

See Figs. 12, 13, 14, 15 and 16.

## Footnotes

1 Total protein concentrations are defined as follows: *P*_*Tot*_=*P*_*C*_+*P*_*CP*_+*P**C*_*C*_+*P**C*_*N*_+*P**C*_*CP*_+*P**C*_*NP*_+*I*_*N*_, *C*_*Tot*_=*C*_*C*_+*C*_*CP*_+*P**C*_*C*_+*P**C*_*N*_+*P**C*_*CP*_+*P**C*_*NP*_+*I*_*N*_, *B*_*Tot*_=*B*_*C*_+*B*_*CP*_+*B*_*N*_+*B*_*NP*_+*I*_*N*_.

2 planor: Generation of regular factorial designs https://CRAN.R-project.org/package=planor

3 multisensi: Multivariate Sensitivity Analysis https://CRAN.R-project.org/package=multisensi
